# m^6^A demethylase ALKBH5 inhibits tumor growth and metastasis by reducing YTHDFs-mediated *YAP* expression and inhibiting miR-107/LATS2–mediated YAP activity in NSCLC

**DOI:** 10.1186/s12943-020-01161-1

**Published:** 2020-02-27

**Authors:** Dan Jin, Jiwei Guo, Yan Wu, Lijuan Yang, Xiaohong Wang, Jing Du, Juanjuan Dai, Weiwei Chen, Kaikai Gong, Shuang Miao, Xuelin Li, Hongliang Sun

**Affiliations:** 1 Clinical Medical Laboratory, Binzhou Medical University Hospital, Binzhou, 256603 People’s Republic of China; 2 Cancer research institute, Binzhou Medical University Hospital, Binzhou, 256603 People’s Republic of China; 3 Department of Thyroid and Breast Surgery, Binzhou Medical University Hospital, Binzhou, 256603 People’s Republic of China; 4 Department of reproductive medicine, Binzhou Medical University Hospital, Binzhou, 256603 People’s Republic of China

**Keywords:** ALKBH5, YTHDFs, HuR, miR-107, LATS2, YAP

## Abstract

**Background:**

The importance of mRNA methylation erased by ALKBH5 in mRNA biogenesis, decay, and translation control is an emerging research focus. Ectopically activated YAP is associated with the development of many human cancers. However, the mechanism whereby ALKBH5 regulates YAP expression and activity to inhibit NSCLC tumor growth and metastasis is not clear.

**Methods:**

Protein and transcript interactions were analyzed in normal lung cell and NSCLC cells. Gene expression was evaluated by qPCR and reporter assays. Protein levels were determined by immunochemical approaches. Nucleic acid interactions and status were analyzed by immunoprecipitation. Cell behavior was analyzed by standard biochemical tests. The m^6^A modification was analyzed by MeRIP.

**Results:**

Our results show that YAP expression is negatively correlated with ALKBH5 expression and plays an opposite role in the regulation of cellular proliferation, invasion, migration, and EMT of NSCLC cells. ALKBH5 reduced m^6^A modification of *YAP*. YTHDF3 combined *YAP* pre-mRNA depending on m^6^A modification. YTHDF1 and YTHDF2 competitively interacted with YTHDF3 in an m^6^A-independent manner to regulate *YAP* expression. YTHDF2 facilitated *YAP* mRNA decay via the AGO2 system, whereas YTHDF1 promoted *YAP* mRNA translation by interacting with eIF3a; both these activities are regulated by m^6^A modification. Furthermore, ALKBH5 decreased YAP activity by regulating miR-107/LATS2 axis in an HuR-dependent manner. Further, ALKBH5 inhibited tumor growth and metastasis in vivo by reducing the expression and activity of YAP.

**Conclusions:**

The presented findings suggest m^6^A demethylase ALKBH5 inhibits tumor growth and metastasis by reducing YTHDFs-mediated YAP expression and inhibiting miR-107/LATS2–mediated YAP activity in NSCLC. Moreover, effective inhibition of m^6^A modification of ALKBH5 might constitute a potential treatment strategy for lung cancer.

## Background

Non-small cell lung cancer (NSCLC) is a highly malignant tumor, with high clinical incidence and mortality that annually increase globally [1]. Even when individuals with NSCLC are treated with a combination of surgery and chemotherapy, the survival rate is low because of cancer cell metastasis and invasion [2]. Therefore, finding an effective target to inhibit cancer cell growth and invasion in NSCLC is urgently needed.

More than 100 chemical modifications of cellular RNA are known. Reversible RNA modification constitutes a new level of post-transcriptional regulation of gene expression, involved in many physiological processes and with multiple biological roles [3]. N^6^-methyladenine (m^6^A) is a dynamic reversible chemical modification. It is the most abundant internal modification of mRNA and a new transcription marker. In mammals, plants, and some prokaryotes, m^6^A is widely involved in mRNA metabolism, affecting mRNA stability and splicing, RNA nucleation, RNA-protein interaction, and mRNA translation [4, 5]. The enzymes m^6^A demethylases are also known as “erasers”. Two eukaryotic m^6^A demethylases have been identified to date: fat mass and obesity-associated protein (FTO) and AlkB homolog 5 (ALKHB5). ALKHB5 is a member of the AlkB family, which plays an important role in regulation of many biological processes, such as mRNA processing [6]. According to recent studies, ALKBH5 plays an important role in the occurrence and development of cancer in human [6, 7]. For example, ALKBH5 holds prognostic value and inhibits the metastasis of colon cancer [8]; ALKBH5 controls trophoblast invasion at the maternal-fetal interface by regulating the stability of *Cyr61* mRNA [9]; METTL3 and ALKBH5 oppositely regulate m^6^A modification of *TFEB* mRNA, dictating the fate of hypoxia/reoxygenation-treated cardiomyocyte [10]; ALKBH5 inhibits pancreatic cancer cell motility by decreasing methylation of the long non-coding RNA KCNK15-AS1 [11]. Moreover, HuR restrains translation inhibition mediated by some miRNAs by directly binding and sequestering microRNAs (miRNAs). In addition, studies have shown that m^6^A indirectly impacts transcript stability, by affecting HuR binding and microRNA targeting [12, 13]. However, the mechanism through which ALKBH5 regulates NSCLC tumor growth and metastasis is not clear.

A group of YTH domain-containing proteins (YTHDFs) have been identified as m^6^A “readers” that recognize m^6^A marks and mediate m^6^A function [14]. The human YTH domain family consists of three members: YTHDF1–3. Each member contains a highly conserved single-stranded RNA-binding domain, located at their carboxyl termini (the YTH domain) and a relatively less conserved amino-terminal region [15]. YTHDF1 improves the translation efficiency by binding to m^6^A-modified mRNA [16], whereas YTHDF2 reduces the stability of mRNA by recruiting an mRNA degradation system [17]. YTHDF3 serves as a hub to fine-tune the accessibility of RNA to YTHDF1 and YTHDF2. YTHDFs have many important biological functions [18]. For instance, YTHDF3 suppresses interferon-dependent antiviral responses by promoting FOXO3 translation in HREpiC cells [19] and YTHDF2 promotes lung cancer cell growth by facilitating translation of 6-phosphogluconate dehydrogenase mRNA [20]. However, the manner in which YTHDF3 cooperates with YTHDF1 and YTHDF2 to promote the translation or decay of m^6^A-modified YAP mRNA in NSCLC remains to be elucidated.

MicroRNAs (miRNAs) are a group of non-coding single-stranded RNA molecules 20–24 nucleotides-long, encoded by endogenous genes. miRNA interacts with a specific mRNA, triggering its degradation, inhibiting translation and widely participating in the organism growth, development, differentiation, metabolism, defenses, and other processes [21]. Significant differences in the expression of various miRNAs in healthy cells and tumor cells have been recently reported. These miRNAs play a role similar to that of proto-oncogenes or tumor suppressor genes, by regulating different target genes, and are closely related to the occurrence, development, treatment, and prognosis of tumors in human [22].

The Hippo signaling pathway is an inhibitory pathway that hinders cell growth and controls cell proliferation, organ size, and homeostasis [23]. This pathway is highly evolutionarily conserved. The main components of the mammalian pathway are Mst1/2, LATS1/2, and Yap/TAZ. After activation of the Hippo signaling pathway, Mst1/2, as the core component of this kinase chain, is activated and phosphorylates a downstream component of LATS1/2. LATS1/2 mainly inhibits the proliferation and migration of tumor cells by blocking cell cycle progression and plays an important regulatory role in cell apoptosis. LATS1/2 phosphorylates Yap/TAZ, which inhibits YAP activity; then, cytoplasmic 14–3-3 protein binds phosphorylated Yap to promote YAP degradation. Dephosphorylated Yap/TAZ translocate to the nucleus, where it binds tea domain family members (TEADs) to promotes target gene transcription. The target genes include *Ctgf*, *Cyr61*, *Oct4*, *p73*, and *ZEB1*, and control organ size, tumor cell proliferation, and metastasis [24]. However, the transcriptional regulation of the Hippo signaling pathway, especially following *YAP* transcription modified by m^6^A, has not yet been delineated.

We here aimed to explore the mechanism whereby ALKBH5 inhibits tumor growth and metastasis by regulating the expression and activity of YAP in NSCLC. Using lung healthy cells and tumor cells, we demonstrated the following: (1) ALKBH5 decreases the level of m^6^A *YAP* mRNA modification; (2) YTHDF1 and YTHDF2 competitively bind YTHDF3 in an m^6^A-independent manner to regulate *YAP* expression; (3) YTHDF2 facilitates the decay of *YAP* mRNA, which is mediated by Argonaute 2 (AGO2) system and regulated by m^6^A modification; (4) YTHDF1 promotes m^6^A modification-mediated *YAP* mRNA translation via interaction with eIF3a; (5) ALKBH5 decreases the activity of YAP by regulating the miR-107/LATS2 axis in an HuR-dependent manner. These findings clarify the role of m^6^A signaling in the control of YAP expression and activity and suggest novel prognostic factors for NSCLC tumor growth and metastasis.

## Methods

### Molecular biology

Myc-tagged YAP, YTHDF1, YTHDF2, YTHDF3 and Flag-tagged ALKBH5, YTHDF1, YTHDF2, YTHDF3 constructs were made using the pcDNA 3.1 vector (Invitrogen, Carlsbad, CA, USA). Sequences encoding the Myc epitope (EQKLISEEDL) and Flag epitope (DYKDDDDK) were added by PCR through replacement of the first Met-encoding codon in the respective cDNA clones.

### Cell lines and culture

Human lung normal cell line BEAS-2B and NSCLC cell lines A549, H1299, Calu6 and H520 were purchased from American Type Culture Collections (Manassas, VA). Cell lines were cultivated in RPMI-1640 medium supplemented with 10% FBS (Hyclone, USA), penicillin/streptomycin (100 mg/mL). Culture flasks were kept at 37 °C in a humid incubator with 5% CO_2_.

### RNA isolation and reverse transcription (RT)-PCR assay

We used TRIzol reagent (TransGen Biotech, Beijing, China) to isolate total RNA from the samples. RNA was reverse transcribed into first-strand cDNA using a TransScript All-in-One First-Strand cDNA Synthesis Kit (TransGen Biotech). cDNAs were used in RT-PCR and quantitative real-time PCR assay with the human GAPDH gene as an internal control. The final quantitative real-time PCR reaction mix contained 10 μL Bestar® SYBR Green qPCR Master Mix, Amplification was performed as follows: a denaturation step at 94 °C for 5 min, followed by 40 cycles of amplification at 94 °C for 30 s, 58 °C for 30 s and 72 °C for 30 s. The reaction was stopped at 25 °C for 5 min. The relative expression levels were detected and analyzed by ABI Prism 7900HT/FAST (Applied Biosystems, USA) based on the formula of 2^-ΔΔct^. RT-PCR analysis of miR-107 was conducted with One step miRNA RT kit (D1801, HaiGene, China) for reverse-transcription. The PCR was performed with the Dream taq Green master mix (Fermentas, K1082) following the manufacturer’s protocols, then used the 4% agarose gel at 120 V for 70 min. We got the images of RT-PCR by Image Lab™ Software (ChemiDocTM XRS+, BiO-RAD) and these images were TIF with reversal color format. Primers for qPCR:

ALKBH5 forward primer: 5′-GCCTATTCGGGTGTCGGAAC-3′.

ALKBH5 reverse primer: 5′-CTGAGGCCGTATGCAGTGAG-3′.

YTHDF1 forward primer: 5′-GCACACAACCTCCATCTTCG-3′.

YTHDF1 reverse primer: 5′-AACTGGTTCGCCCTCATTGT-3′.

YTHDF2 forward primer: 5′-TCTGGAAAAGGCTAAGCAGG-3′.

YTHDF2 reverse primer: 5′-CTTTTATTTCCCACGACCTTGAC-3′.

*YTHDF3 forward primer:* 5′-TGACAACAAACCGGTTACCA-3′.

*YTHDF3 reverse primer:* 5′-TGTTTCTATTTCTCTCCCTACGC-3′.

YAP forward primer: 5′-GGATTTCTGCCTTCCCTGAA-3′.

YAP reverse primer: 5′-GATAGCAGGGCGTGAGGAAC-3′.

Cyr61 forward primer: 5′-GGTCAAAGTTACCGGGCAGT-3′.

Cyr61 reverse primer: 5′-GGAGGCATCGAATCCCAGC-3′.

CTGF forward primer: 5′-ACCGACTGGAAGACACGTTTG-3′.

CTGF reverse primer: 5′-CCAGGTCAGCTTCGCAAGG-3′.

HuR forward primer: 5′-CGGAATTCAATACAATGTCTAATGG-3′.

HuR reverse primer: 5′-GGGGTACCATTGGCGCAAAATGAG-3′.

LATS2 forward primer: 5′-CAGGATGCGACCAGGAGATG-3′.

LATS2 reverse primer: 5′-CCGCACAATCTGCTCATTC-3′.

E-cadherin forward primer: 5′-ACCATTAACAGGAACACAGG − 3′.

E-cadherin reverse primer: 5′-CAGTCACTTTCAGTGTGGTG-3′.

Vimentin forward primer: 5′- CGCCAACTACATCGACAAGGTGC-3′.

Vimentin reverse primer: 5′-CTGGTCCACCTGCCGGCGCAG-3′.

GAPDH forward primer: 5′-CTCCTCCTGTTCGACAGTCAGC-3′.

GAPDH reverse primer: 5′-CCCAATACGACCAAATCCGTT-3′.

miR-107 forward primer: 5′- GCCGAATTCAAAGCGAGATTCCATCAGCA-3′.

miR-107 reverse primer: 5′- GCCGGATCCTGTCAACCCAGAACTCAAAGG-3′.

### Western blot analysis

Human lung cancer cells were transfected with the relevant plasmids and cultured for 48 h. For western blot analysis, cells were lysed in NP-40 buffer (10 mM Tris pH 7.4, 150 mM NaCl, 1% Triton X-100, 1 mM EDTA pH 8.0, 1 mM EGTA pH 8.0, 1 mM PMSF, and 0.5% NP-40) at 25 °C for 40 min. The lysates were added to 5× loading dye and then separated by electrophoresis. The primary antibodies used in this study were 1:1000 rabbit anti-Flag (sc-166,384, Santa Cruz, Dallas, TX, USA) and 1:1000 Abcam (Cambridge, UK) antibody of Myc (ab32072), ALKBH5 (ab234528), YAP (ab56701), YTHDF1 (ab220162), YTHDF2 (ab220163), YTHDF3 (ab220161), HuR (ab200342), LATS2 (ab135794), CTGF (ab6992), Cyr61 (ab24448), AGO2 (ab32381), eIF3a (ab86146), Vimentin (ab45939), E-cadherin (ab1416), cleaved Capase-3 (ab32042) and Tubulin (ab6046).

### Immunohistochemical analysis

Tumor tissues were fixed in 4% paraformaldehyde overnight and then embedded in paraffin wax. Four-micrometer thick sections were stained using hematoxylin and eosin (H&E) for histological analysis.

### Over-expression and knockdown of genes

Overexpressing plasmid (2 μg), siRNA (1.5 μg) and shRNA (1.5 μg) of indicated genes were transfected into cells using Lipofectamine 2000 (Invitrogen, Carlsbad, CA) for over-expression and knockdown of indicated genes, followed by analysis 48–72 h later. The selected sequences for knockdown as follow:

shALKBH5–1: 5′-CCTCATAGTCGCTGCGCTCG-3′.

shALKBH5–2: 5′-ATAGTTGTCCCGGGACGTCA-3′.

siYAP-1: 5′- AAGGTGATACTATCAACCAAA-3′.

siYAP-2: 5′- AAGACATCTTCTGGTCAGAGA-3′.

siALKBH5–1: 5′-ACAAGTACTTCTTCGGCGA-3′.

siALKBH5–2: 5′-GCGCCGTCATCAACGACTA-3′.

siYTHDF1–1: 5′-CCGCGTCTAGTTGTTCATGAA-3′.

siYTHDF1–2: 5′-CCTCCACCCATAAAGCATA-3′.

siYTHDF2–1: 5′-CTGCCATGTCAGATTCCTA-3′.

siYTHDF2–2: 5′-GCTCCAGGCATG AATACTATA-3′.

siYTHDF3–1: 5′-GGACGTGTGTTTATAATTA-3′.

siYTHDF3–2: 5′-GACTAGCATTGCAACCAAT-3′.

siAGO2: 5′-GCACGGAAGTCCATCTGAA-3′.

sieIF3a: 5′-CAGTTGATGGCAAATTACT-3′.

siHuR: 5′-TGTCAAACCGGATAAACGC-3′.

sicontrol: 5′-TTCTCCGAACGTGTCACGA-3′.

shcontrol: 5′-ACGTGACACGTTCGGAGAATT-3′.

### CCK8 assays

Cell viability and growth was determined using CCK8 assays in 96-well plates. Cells were transfected with the relevant plasmids culturing for 48 h, followed by incubation with 10 μL CCK8 for 4 h. Absorbance was read at 450 nm using a spectrophotometer (Tecan, Männedorf, Switzerland).

### Immunofluorescent staining

To examine the protein expression and location by immunofluorescent staining, NSCLC cells were seeded onto coverslips in a 24-well plate and left overnight. Cells were then fixed using 4% formaldehyde for 30 min at 25 °C and treated with 3% bovine serum albumin (BSA) in phosphate buffered saline (PBS) for 30 min. The coverslips were incubated with rabbit anti-ALKBH5, YTHDF1, YTHDF2, YTHDF3, YAP, CTGF, Cyr61, Ki67, Annexin V, Ki67, Edu, Vimentin and mouse anti-E-cadherin monoclonal antibody (Abcam) at 1:200 dilution in 3% BSA. Alexa-Fluor 488 (green, 1:500, A-11029; Invitrogen, USA) and 594 (red, 1:500, A-11032; Invitrogen, USA) tagged anti-rabbit or -mouse monoclonal secondary antibody at 1:1000 dilution in 3% BSA. Hoechst (3 μg/mL, cat. no. E607328; Sangon Biotech Co., Ltd.) was added for nuclear counterstaining. Six pictures were obtained with a Zeiss Axio Imager Z1 Fluorescent Microscope (Zeiss, Oberkochen, Germany). The results were presented as the Mean ± SD.

### Subcellular fraction

Transfected A549 and H1299 cells were harvest in PBS and resuspended for 10 min on ice in 500 μL CLB Buffer (10 mM Hepes, 10 mM NaCl, 1 mM KH_2_PO_4_, 5 mM NaHCO_3_, 5 mM EDTA, 1 mM CaCl_2_, 0.5 mM MgCl_2_). Thereafter, 50 μL of 2.5 M sucrose was added to restore isotonic conditions. The first round of centrifugation was performed at 6300 g for 5 min at 4 °C. The pellet washed with TSE buffer (10 mM Tris, 300 mM sucrose, 1 mM EDTA, 0.1% NP40, PH 7.5) at 1000 g for 5 min at 4 °C until the supernatant was clear. The resulting pellets were nucleus. The resulting supernatant from the first round was transferred and subjected to differential centrifugation at 14000 rpm for 30 min. The resulting pellets were membranes and the supernatant were cytoplasm.

### RNA immunoprecipitation assay

RNA immunoprecipitation (RIP) was performed using Magna RIP™ RNA-Binding Protein Immunoprecipitation Kit (Millipore) according to the manufacturer′s instructions. Briefly, cells were collected and lysed in complete RIPA buffer containing a protease inhibitor cocktail and RNase inhibitor. Next, the cell lysates were incubated with RIP buffer containing magnetic bead conjugated with indicated antibody (Abcam) or control normal human IgG. The samples were digested with proteinase K to isolate the immunoprecipitated RNA. The purified RNA was finally subjected to qPCR to demonstrate the presence of the binding targets.

### RNA pulldown

Harvested cells were rinsed and sonicated in NET-2 buffer (50 mM Tris-HCl, pH 7.4, 150–300 mM NaCl, 0.05% NP40, PMSF, Benzamidine). Cell lysates were incubated with biotin-labeled probes synthesized from Sangon (Shanghai, China), and then pulled down with streptavidin beads (Sigma-Aldrich). Precipitated RNA and proteins were subsequently subjected to RT-PCR and western blot analyses, respectively.

### MS2 coat protein system to enrich mRNA

The MS2 coat protein system was performed as described previously [25]. Briefly, stably expressed pcDNA3.1-YAP-MS2–12X (YAP-MS2) NSCLC cells were co-transfected with pcDNA3.1-MS2-GFP and relevant pcDNA3.1-YTHDFs then cultivated in RPMI-1640 medium supplemented with 10% FBS (Hyclone, USA), penicillin/streptomycin (100 mg/mL). Culture flasks were kept at 37 °C for 48 h in a humid incubator with 5% CO_2_. The cell lysate from these transfected cells were immunoprecipitated by GFP antibody to enrich YAP mRNA then performed the following experiment.

### Total m^6^A measurement

The total m^6^A content of Total RNA was determined using an m^6^A methylation quantification kit (EpiGentek, USA). Briefly, after total RNA was isolated and purified, the bind RNA was planted to the assay wells and cultured with the capture antibody. After that, the wells were washed, and the detection antibody and enhancer solution were added. The m^6^A level was detected according to the fluorescence after the wells were incubated with the fluoro developer solution.

### RNA m^6^A quantification using HPLC–tandem mass spectrometry

mRNA was isolated from total RNA by using a Dynabeads mRNA Purification Kit (Thermo Fisher Scientific), and rRNA contaminants were removed by using a RiboMinus Eukaryote Kit (Thermo Fisher Scientific). Subsequently, mRNA was digested into nucleosides by using nuclease P1 and alkaline phosphatase and was then filtered with a 0.22 mm filter. The amount of m^6^A was measured according to HPLC–tandem mass spectrometry, following the published procedure [26]. Quantification was performed by using the standard curve obtained from pure nucleoside standards that were run with the same batch of samples. The ratio of m^6^A to A was calculated based on the calibrated concentrations.

### m^6^A mRNA immunoprecipitation

m^6^A Ribonucleoprotein Immunoprecipitation reactions were performed by first isolating PolyA+ RNA from treated NSCLC cells. Protein G Dynabeads (Thermo Fisher Scientific, Baltics UAB) were washed 3× in 1 mL of IPP buffer (10 mM Tris-HCL pH 7.4, 150 mM NaCl, 0.1% NP-40). 25 μl of beads required per IP. Anti-N^6^-methyladenosine human monoclonal antibody (EMD Millipore, Temecula, CA, MABE1006) was added to the beads (5 μg/IP) and brought up to 1 mL with IPP buffer. Bead mixture was tumbled for 16 h at 4 °C. Beads were washed 5× with IPP buffer and 100 ng of PolyA+ RNA was added to the beads along with 1 mM DTT and RNase out. The mixture was brought up to 500 μl with IPP buffer. Bead mixture was tumbled at 4 °C for 4 h. Beads were washed 2× in IPP buffer, placed into a fresh tube, and washed 3× more in IPP buffer. m^6^A RNA was eluted off the beads by tumbling with 125 μl of 2.5 mg/mL N^6^-Methyladenosine-5′-monophosphate sodium salt (CHEM-IMPEX INT’L INC., Wood Dale, IL). Supernatant was added to Trizol-LS followed by RNA isolation as manufacture’s protocol. Final RNA sample was brought up in 10 μl of water.

### qPCR for MeRIP

Reverse transcription was performed on 10 μl m^6^A PolyA+ RNA from the MeRIP with the iScript cDNA synthesis kit (Bio-Rad Laboratories, Hercules, CA). After diluting cDNA two-fold, quantitative real-time PCR was performed using the ABI Prism 7900HT/FAST (Applied Biosystems, USA) and primers from Integrated DNA Technologies, Inc. (Coralville, Iowa). Primers used are listed in the follows. Primer efficiency was verified to be over 95% for all primer sets used. Quantification of mRNA from the MeRIP was carried out via 2^-ΔΔct^. analysis against non-immunoprecipitated input RNA. All real-time PCR primer sets were designed so the products would span at least one intron (> 1 kb when possible), and amplification of a single product was confirmed by agarose gel visualization and/or melting curve analysis. Primers for MeRIP:

YAP m^6^A peak1 Forward primer: 5′-TGCGCGTCGGGGGAGGCAGAAG-3′.

YAP m^6^A peak1 Reverse primer: 5′-GGAATGAGCTCGAACATGCTG-3′.

YAP m^6^A peak2 Forward primer: 5′-TGAACCAGAGAATCAGTCAGAG-3′.

YAP m^6^A peak2 Reverse primer: 5′-GTACTCTCATCTCGAGAGTG-3′.

YAP m^6^A peak3 Forward primer: 5′-CCAGTGTCTTCTCCCGGGATG-3′.

YAP m^6^A peak3 Reverse primer: 5′-TATCTAGCTTGGTGGCAGCC-3′.

### Enzyme linked immunosorbent assay (ELISA) analysis

A549 and H1299 cells were transfected with relevant plasmids and cultured for indicated times. For ELISA analysis, the YAP (Human) Cell-Based ELISA Kit (KA3582, Abnova, Taiwan, China) was used to detect the YAP protein expression following the manufacturer’s protocols. Briefly, fixing Solution, Quenching Buffer, Blocking Buffer, 1x primary antibodies were orderly added into the fixed cells and incubate overnight at 4 °C then add 50 μl of HRP-conjugated secondary antibodies and incubate for 1.5 h at room temperature and add 50 μl of TMB One-Step Reagent and incubate for 30 min at room temperature. Absorbance was read at 450 nm using a spectrophotometer (Tecan, Männedorf, Switzerland).

### Analysis of publicly available datasets

To analyze correlation between ALKBH5, YAP, YTHDF1, YTHDF2 and YTHDF3 expression level and prognostic outcome of patients, Kaplan-Meier survival curves of NSCLC patients with low and high expression of ALKBH5, YAP, YTHDF1, YTHDF2 and YTHDF3 were generated using Kaplan-Meier Plotter (www.kmplot.com/analysis and www.oncolnc.org) [27].

### Human lung cancer specimen collection

All the human lung cancer and normal lung specimens were collected in Affiliated Hospital of Binzhou Medical College with written consents of patients and the approval from the Institute Research Ethics Committee.

### In vivo experiments

To assess the in vivo effects of ALKBH5, 3 to 5-week old female BALB/c athymic (NU/NU) nude mice were housed in a level 2 biosafety laboratory and raised according to the institutional animal guidelines of Binzhou Medical University. All animal experiments were carried out with the prior approval of the Binzhou Medical University Committee on Animal Care. For the experiments, mice were injected with 5 × 10^6^ lung cancer cells with stably expression of relevant plasmids and randomly divided into indicated groups (five mice per group). To assess the in vivo effects of cycloleucine, the xenografted tumors had reached approximately 5 mm in diameter from mice and then these xenografted mice were feed with Vehicle or cycloleucine (25 mg/kg twice weekly) and tumor volume were measured every 3 day. Tumor volume was estimated as 0.5 × a^2^ × b (where a and b represent a tumors short and long diameter, respectively). Mice were euthanized after 7 weeks and the tumors were measured a final time. Tumor and organ tissue were then collected from xenograft mice and analyzed by immunohistochemistry.

### Statistical analysis

Each experiment was repeated at least three times. The statistical analyses of the experiment data were performed by using a two-tailed Student’s paired T-test and one-way ANOVA. Statistical significance was assessed at least three independent experiments and significance was considered at either *P*-value < 0.05 was considered statistically significant and highlighted an asterisk in the figures, while *P*-values < 0.01 were highlighted using two asterisks and P-values < 0.001 highlighted using three asterisks in the figures.

## Results

### Ectopic expression of YAP and ALKBH5 regulates cell proliferation, invasion, migration, and EMT in NSCLC cells

**Fig. 1:**
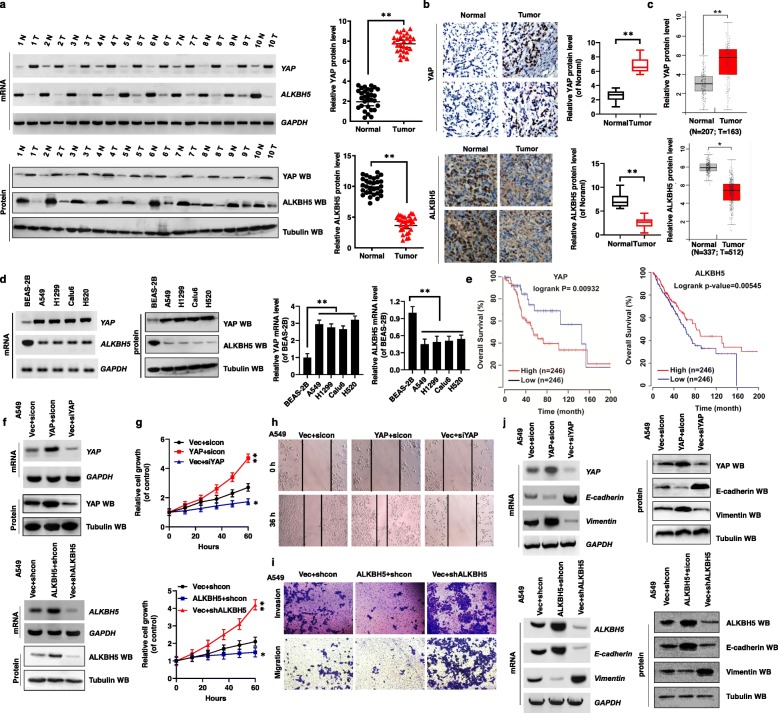
Ectopic expression of YAP and ALKBH5 regulates cell proliferation, invasion, migration, and EMT in NSCLC cells. (**a**) The mRNA and protein levels of YAP and ALKBH5 were analyzed by RT-PCR and western blot assays in the paired fresh NSCLC tumor cancer tissues (Tumor) and matched adjacent normal tissues (Normal) (left panel, *n* = 10; right panel, *n* = 30). (**b**) The expressions of YAP and ALKBH5 were analyzed by immunohistochemical (IHC) assay in the human lung cancer tissues and their normal adjacent lung tissues (n = 5). (**c**) The TCGA database indicated that YAP was higher but ALKBH5 was lower in tumor tissues than their normal tissues. (**d**) The mRNA and protein levels of YAP and ALKBH5 were analyzed by RT-PCR, qPCR and western blot assays in NSCLC cell lines and their control (normal) cell, BEAS-2B. (**e**) High expression of YAP (*P = 0.00932*) but low expression of ALKBH5 (*P = 0.00545*) were associated with worse prognosis for NSCLC patients. (**f-j**) A549 cells were transfected with indicated genes of YAP and ALKBH5. (**f**) The expressions of YAP and ALKBH5 were analyzed by RT-PCR and western blot assays. (**g**) The cellular growth was analyzed by CCK8 assay. (**h**) The migration viability was analyzed by scratch assay. (**i**) The cellular invasion and migration growths were analyzed by transwell assay. (**j**) The expressions of E-cadherin and Vimentin were analyzed by RT-PCR and western blot assays. Results were presented as mean ± SD of three independent experiments. *P < 0.05 or **P < 0.01 indicates a significant difference between the indicated groups

For the study, NSCLC samples were obtained from patients who underwent lung resection surgery at the Affiliated Hospital of Binzhou Medical University (Binzhou, China) between January 2018 and January 2019. The clinicopathological findings of ALKBH5, YAP, YTHDF1 and YTHDF2 expressions in the samples and pathological grades of NSCLC patients are summarized in Table 1 and Additional file 1: Table S1. We found that YAP expression was negatively correlated with ALKBH5 expression in NSCLC tumor tissues (Table 1). Interestingly, opposite trends of YAP and ALKBH5 expression were observed in paired fresh NSCLC tumor cancer tissues (Tumor) and matched adjacent normal tissues (Normal) by RT-PCR, western blotting, and qPCR (left panel, *n* = 10; right panel, *n* = 30) (Fig. 1a, Additional file 2: Fig. S1a, b, n = 10), and immunohistochemistry (IHC) (Fig. 1b, n = 10). Concurrently, TCGA database (https://www.cancer.gov) analysis revealed similar results (Fig. 1c). We also found that the mRNA and protein levels of YAP were higher, while those of ALKBH5 were lower, in NSCLC cancer cells than in the control (normal) cells BEAS-2B (Fig. 1d, Additional file 2: Fig. S1c). Next, publicly available datasets were screened and used to determine the prognostic correlation between YAP/ALKBH5 expression and NSCLC patient survival [27]. Results show that the overall survival (OS) time of patients with high YAP expression (*n* = 246) was shorter than that of patients with low YAP expression (n = 246) (*P = 0.00932*) whereas OS time of patients with low ALKBH5 expression (n = 246) was shorter than that of patients with high ALKBH5 expression (n = 246) (*P = 0.00545*) [27](Fig. 1e). These observations suggest that deregulated expression of YAP and ALKBH5 is closely associated with the occurrence and development of NSCLC.

**Table 1:** Patient’s demographics and tumor characteristics and association of ALKBH5 levels with clinicopathological features

Characteristics	No. of patients, *N* = 60 (%)	*P* value
Patients Parameter		
Age (years)		0.871
Average [range]	53 [30–81]	
< 53	20 (33.3)	
≥ 53	40 (66.7)	
Gender		0.714
Male	32 (53.3)	
Female	28 (46.7)	
Smoking history		
Smoker	35 (58.3)	0.12
Non-smoker	25 (41.7)	
Tumor Characteristics		
Tumor size (cm)		0.009**
< 4	10 (16.7)	
≥ 4	50 (83.3)	
Differentiation		0.044*
Poor	41 (68.3)	
Well-moderate	19 (31.7)	
Lymph node metastasis		0.014*
N-	9 (15.0)	
N+	51 (85.0)	
Distant metastasis		0.008**
M-	8 (13.3)	
M+	52 (86.7)	
Expression of ALKBH5		
Protein level		
high	2 (3.3)	0.086
median	8 (13.3)	0.078
low	50 (83.4)	0.002**
mRNA level		
high	3 (5.0)	0.074
median	9 (15.0)	0.063
low	48 (80.0)	0.001**
Expression of YAP		
Protein level		
high	46 (76.6)	0.008**
median	9 (15.5)	0.062
low	5 (7.9)	0.865
mRNA level		
high	48 (80.0)	0.005**
median	8 (13.3)	0.084
low	4 (6.7)	0.754

Differences between experimental groups were assessed by Student’s t-test or one-way analysis of variance. Data represent mean ± SD. *p < 0.05; **p < 0.01

To investigate the function of YAP and ALKBH5 in NSCLC cells, we then used siRNAs and shRNAs to knock down *YAP* (siYAP-1 and siYAP-2) and *ALKBH5* (shALKBH5–1 and shALKBH5–2), or overexpressed these genes by transfecting A549 and H1299 cells with a plasmid encoding Myc-YAP or Flag-ALKBH5, accordingly (Fig. 1f, Additional file 2: Fig. S1d, e). Knocking down *YAP* using siYAP-2 and A*LKBH5* using shALKBH5–1 was more effective than siYAP-1 and shALKBH5–2 (Additional file 2: Fig. S1f, g). Therefore, siYAP-2 and shALKBH5–1 were used in subsequent knockdown experiments. CCK8 analysis revealed that overexpression of *YAP* and knockdown of *ALKBH5* enhanced cellular growth and viability, while knockdown of *YAP* and overexpression of *ALKBH5* reduced cellular growth and viability (Fig. 1g, Additional file 2: Fig. S1h, i). Ki67/Edu (commonly used as a proliferation indicator) staining (Additional file 2: Fig. S1j, k), analysis of the protein levels of cleaved caspase 3 (commonly used as an apoptosis indicator) (Additional file 2: Fig. S1l, m), and clone formation assays (Additional file 3: Fig. S2a) confirmed these findings. Similar results for cellular migration and invasion growth were obtained with A549 and H1299 cells, as detected by scratch and transwell assays (Fig. 1h, i, Additional file 3: Fig. S2b-e). Further, to assess the role of YAP and ALKBH5 in EMT, we evaluated variations in E-cadherin and Vimentin levels associated with the YAP and ALKBH5 status. RT-PCR, western blotting, and immunofluorescence analyses showed that *YAP* silencing and *ALKBH5* overexpression decreased mRNA and protein levels of Vimentin, and increased those of E-cadherin in A549 and H1299 cells; the opposite result was observed upon *YAP* overexpression and *ALKBH5* silencing (Fig. 1j, Additional file 3: Fig. S2f, g). Furthermore, spearman rank correlation analysis of data in the TCGA database revealed positive correlations between YAP and Vimentin levels, and ALKBH5 and E-cadherin levels, but negative correlations between YAP and E-cadherin levels, and ALKBH5 and Vimentin levels (Additional file 3: Fig. S2h, i). These observations indicated that YAP expression is negatively correlated with ALKBH5 expression, and that ectopic expression of YAP and ALKBH5 regulates cellular proliferation, invasion, migration, and EMT in NSCLC cells.

Furthermore, to explore ALKBH5 reduction of cell growth, migration and EMT via regulation of YAP, A549 and H1299 cells were solely transfected with ALKBH5 or co-transfected with ALKBH5 and YAP (Additional file 4: Fig. S3a). Data analysis showed that cell viability was increased in A549 and H1299 cells with co-transfection with ALKBH5 and YAP compared to the transfection with ALKBH5 alone (Additional file 4: Fig. S3b). Moreover, similar results for clone formation (Additional file 4: Fig. S3c) and migration (Additional file 4: Fig. S3d) were observed in A549 and H1299 cells with the same treatment. The EMT was increased in A549 and H1299 cells co-transfected with ALKBH5 and YAP compared to the ALKBH5 only transfection by detecting the EMT-related marker, E-cadherin and Vimentin, respectively (Additional file 4: Fig. S3e). These findings indicate that ALKBH5 decreases cell growth, migration and EMT via regulation of YAP in A549 and H1299 cells.

### ALKBH5 controls YAP expression by regulating m^6^A levels in NSCLC

**Fig. 2:**
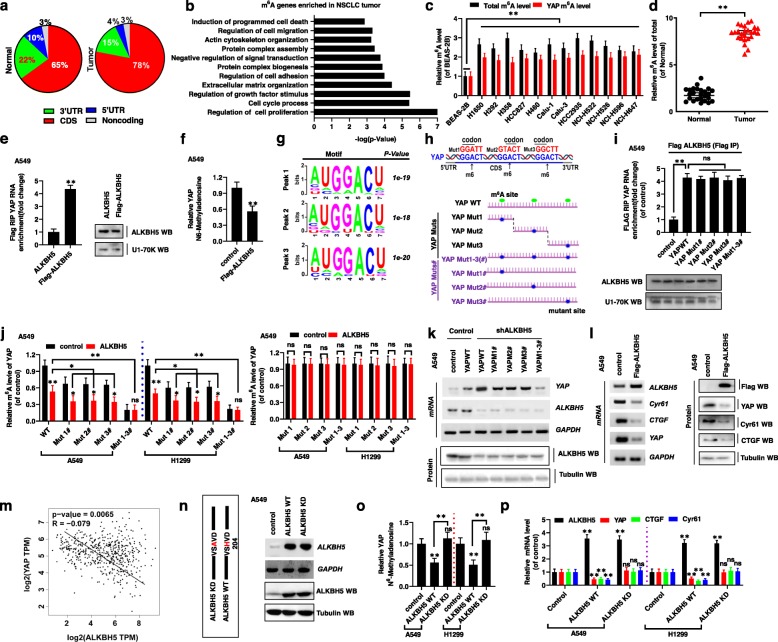
ALKBH5 controls YAP expression by regulation m^6^A level in NSCLC. (**a**) Pie chart depicting the fraction of m^6^A peaks in 4 transcript segments. (**b**) Gene ontology (GO) enrichment analysis of tumor methylated genes in NSCLC patients. (**c**) The m^6^A levels of total and YAP were detected in human NSCLC cell lines determined by m^6^A methylation quantification kit and MeRIP-qPCR assay. (**d**) The total m^6^A levels were higher in 30 paired fresh NSCLC tumor cancer tissues (Tumor) than matched adjacent normal tissues (Normal) (*n* = 30). (**e**) The interaction between ALKBH5 and YAP pre-mRNA was detected by RIP. (**f**) The relative m^6^A level of YAP pre-mRNA was detected by MeRIP-qPCR in A549 cells. (**g**) Sequence motifs in m^6^A peaks identified by using m6Avar database from YAP CDS. (**h**) Putative m^6^A modification sites in the CDS sequence of YAP and synonymous mutations in the YAP CDS. (**i**) The interaction between ALKBH5 and YAP pre-mRNA was detected by RIP. (**j**) The relative of m^6^A level in YAP from co-expression of ALKBH5 and YAP WT/Muts# (left panel) or ALKBH5 and YAP Muts (right panel) in A549 and H1299 cells. (**k**) The relative mRNA levels of YAP were analyzed by RT-PCR. (**l**) The mRNA and protein levels of ALKBH5, YAP, CTGF and Cyr61 were detected in A549 cells. (**m**) The negative correlation between ALKBH5 and YAP was analyzed from TCGA database. (**n**) The expressions of wild type (ALKBH5 WT) and catalytic mutant (ALKBH5 KD) of ALKBH5 were analyzed by RT-PCR and western blot assays. (**o**) The relative of m^6^A level in YAP from ALKBH5 WT or KD transfected A549 cells. (**p**) The mRNA levels of ALKBH5, YAP, CTGF and Cyr61 were detected in A549 and H1299 cells. Results were presented as mean ± SD of three independent experiments. *P < 0.05 or **P < 0.01 indicates a significant difference between the indicated groups. ns, not significant

Recently, m^6^A modification of mRNA was shown to cause many tumors and diseases in mammals by regulating cell differentiation, tissue development, and tumorigenesis [28]. Therefore, we next explored whether m^6^A was involved in the occurrence and development of NSCLC. To verify the specific occurrence of m^6^A in transcripts in NSCLC cancer tissues and normal adjacent tissues, m^6^A peaks were analyzed by HPLC-tandem mass spectrometry, and categorized based on the annotation of gene and non-overlapping segments, as shown in Fig. 2a. Gene ontology enrichment analysis revealed that the m^6^A genes enriched in tumors were primarily involved in the regulation of cell proliferation, growth factor stimulus, and cell cycle process, i.e., the same phenomena that were affected by *YAP* overexpression [29] (Fig. 2b). We also observed that the m^6^A modification of total RNA (Fig. 2c, d), especially *YAP* m^6^A levels (Fig. 2c, Additional file 5: Fig. S4a), was higher in NSCLC tumor cancer tissues and cell lines than in their matched adjacent normal tissues and the control cells BEAS-2B. These findings revealed that m^6^A modification of *YAP* plays a major role in the occurrence and development of NSCLC. Importantly, RNA immunoprecipitation (RIP) assay revealed that *YAP* pre-mRNA interacted with ALKBH5 in A549 and H1299 cells (Fig. 2e, Additional file 5: Fig. S4b). Further, *ALKBH5* overexpression and knockdown decreased or increased the levels of m^6^A modification of *YAP* pre-mRNA, respectively, compared with modification levels of control vectors of ALKBH5 in A549 and H1299 cells (Fig. 2f, Additional file 5: Fig. S4c). Similar results were obtained in A549 and H1299 cells for another m^6^A demethylase, FTO (Additional file 5: Fig. S4d). These findings indicate that ALKBH5 decreases the m^6^A levels on *YAP* pre-mRNA in NSCLC.

MeRIP combined with high throughput sequencing analysis of lung cancer tissues and normal adjacent lung tissues yielded m^6^A peaks within thousands of coding transcripts with high confidence (http://m6avar.renlab.org/). Consensus motif search identified GGACU sequence (Fig. 2g), which a consensus methylation motif reported by others [30]. We have identified three potential consensus motifs in CDS of *YAP* (Fig. 2h, upper panel). To investigate the role of these motifs regulated by ALKBH5, we introduced a synonymous mutation at the putative m^6^A sites in the *YAP* coding region, as follows: YAP Muts series: YAP Mut1, YAP Mut2, and YAP Mut3 (fragments containing only one potential m^6^A site, which was mutated to form YAP Mut1/2/3, respectively); YAP Muts# series: YAP Mut1#, YAP Mut2#, and YAP Mut3# (fragments containing three potential m^6^A sites, wherein only one m^6^A site was mutated to form YAP Mut1#/2#/3#, respectively); and YAP Mut1–3(#) (fragment with three potential m^6^A sites mutated to form YAP Mut1–3(#)) (Fig. 2h, lower panel) [25]. Importantly, the RIP assay revealed that *YAP*-WT and *YAP*-Muts# pre-mRNAs both interacted with ALKBH5, indicating that the interaction between *YAP*-Muts# pre-mRNA and ALKBH5 was not affected by the mutation and ruled out the possibility that YAP-Muts# cannot directly interact with ALKBH5 to influence the detection of YAP m^6^A modification by ALKBH5 (Fig. 2i). We then evaluated the levels of *YAP* m^6^A modification in A549 and H1299 cells co-transfection with *ALKBH5* and the *YAP* mutant genes by using meRIP-qPCR. As hypothesized, ALKBH5 decreased the m^6^A modifications of YAP WT and YAP Muts# (Mut1#, Mut2# and Mut3#) in A549 and H1299 cells compared to the ALKBH5 control vector. However, the reduction level of m^6^A modification was repressed in YAP Muts# compared to YAP WT due to the presence of m^6^A site mutation in YAP Muts#. Importantly, the m^6^A modification of YAP Mut1–3#, containing all m^6^A site mutation, was not decreased in A549 and H1299 cells with overexpression of ALKBH5 compared to ALKBH5 control vector (Fig. 2j, left panel). Moreover, the m^6^A modifications of YAP WT and Muts# (Mut1#, Mut2# and Mut3#) were increased in A549 and H1299 cells with transfection with shALKBH5 compared to ALKBH5 control vector. However, the m^6^A modification of YAP Mut1–3# was unchanged in A549 and H1299 cells with transfection of shALKBH5 compared to ALKBH5 control vector, due to the presence of all m^6^A site mutations in YAP Mut1–3# (Additional file 5: Fig. S4e, left panel). Interestingly, the m^6^A modifications of YAP were unchanged in YAP Muts-transfected A549 and H1299 cells with co-transfection of ALKBH5 or shALKBH5 compared to ALKBH5 control vector, since that the only m^6^A modification site of YAP Muts was mutated (Fig. 2j, right panel, Additional file 5: Fig. S4e, right panel). This indicated that the predicted sites were indeed modified by m^6^A. These findings suggested that ALKBH5 directly interacts with *YAP* pre-mRNA and that this interaction decreases the level of m^6^A modification on *YAP* pre-mRNA.

To further investigate how m^6^A modification affects *YAP* expression, *YAP* mRNA and protein levels were evaluated in A549 and H1299 cells. We found that *YAP* mRNA and protein levels were higher in cells co-transfected with shALKBH5 and *YAP*-WT than in cells co-transfected with shALKBH5 and *YAP*-Muts# (Fig. 2k, Additional file 5: Fig. S4f). Additionally, YAP mRNA and protein levels, as well as the levels of YAP target genes, including *CTGF* and Cyr61, were significantly decreased in A549 and H1299 cells with ectopic *ALKBH5* expression; the opposite was observed in cells upon shALKBH5 expression (Fig. 2l, Additional file 5: Fig. S4g-j). Further, IHC staining revealed that YAP protein levels were higher in tissues with low ALKBH5 expression (Additional file 5: Fig. S4k, l). TCGA database analysis revealed that *ALKBH5* expression was negatively correlated with *YAP* expression (Fig. 2m). This suggested that ALKBH5 decreased YAP expression by regulating m^6^A level in NSCLC. We also performed tethering experiments using plasmids encoding either wild-type or a dominant catalytic variant (ALKBH5 KD: H204A) of ALKBH5 (Fig. 2n, left panel). The mRNA and protein levels of ALKBH5 KD were unchanged compared with those of ALKHB5 WT, which indicated that the mutation of ALKBH5 did not affect the expression of ALKBH5 (Fig. 2n, right panel, Additional file 5: Fig. S4m). Further, the interaction between *YAP* pre-mRNA and ALKBH5 KD was not affected by the substitution (Additional file 5: Fig. S4n). The m^6^A levels of *YAP* (Fig. 2o), and mRNA levels of *YAP*, *CTGF*, and *Cyr61* (Fig. 2p) were insignificantly changed in A549 and H1299 cells transfected with plasmid encoding ALKBH5 KD compared with the control vector. These indicated that ALKBH5 directly binds to YAP pre-mRNA, decreases its m^6^A modification and reduces the expressions of YAP, CTGF and Cyr61, through which need the ALKBH5 catalytic activity in NSCLC.

### YTHDF1 and YTHDF2 competitively bind YTHDF3 in an m^6^A-independent manner to regulate YAP expression

**Fig. 3:**
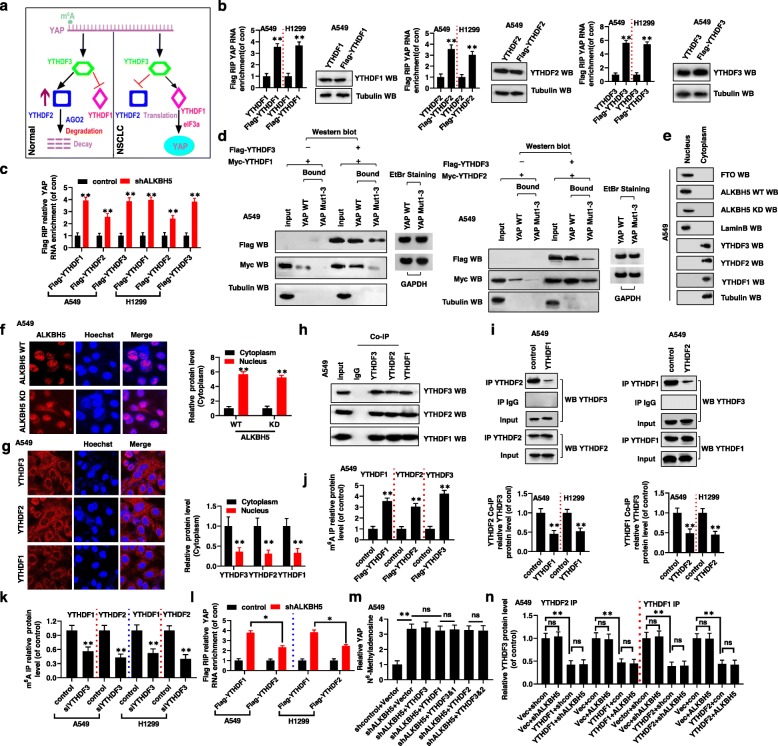
YTHDF1 and YTHDF2 competitively interacted with YTHDF3 in an m^6^A- independent manner to regulate YAP expression. (**a**) The diagram of that YTHDF1 and YTHDF2 competitively interacted with YTHDF3 to regulate YAP expression in NSCLC. (**b, c**) The interaction between YTHDF1/2/3 and YAP mRNA was determined by RIP assay. (**d**) The interactions between YTHDF1/YTHDF2 and the mRNA of YAP were increased when YTHDF3 is existed determined by RNA pulldown assay. (**e**) Western blot analysis indicated that FTO and ALKBH5 were only in nuclear fractions but YTHDF1/2/3 were only in cytoplasm fractions in A549 cells. (**f, g**) Immunofluorescent staining showed that ALKBH5 WT/KD were only in nuclear fractions (**f**) but YTHDF1/2/3 were only in cytoplasm fractions (**g**) in A549 cells. (**h**) Co-IPs performed using lysates collected from A549 cells with immunoprecipitation by either YTHDF1, YTHDF2 or YTHDF3 antibodies. (**i**) The protein level of YTHDF3 was analyzed in lysates collected from either YTHDF1 or YTHDF2 transfected A549 and H1299 cells determined by Co-IPs assays by immunoprecipitation with YTHDF2 or YTHDF1 antibodies, respectively. (**j, k**) The interactions between YTHDF1/2/3 and YAP mRNA were detected in A549 and H1299 cells with transfection with indicated genes determined by Co-IP assay using the MS2 coat protein system. (**l**) The interactions between YTHDF1/2 and YAP mRNA was determined by RIP assay in A549 and H1299 cells with transfection of indicated genes. (**m**) The m^6^A levels of YAP were analyzed by in A549 and H1299 cells with transfection into indicated genes. (**n**) The protein level of YTHDF3 was detected in lysates collected from A549 cells determined by Co-IP assay with immunoprecipitation with either YTHDF1 or YTHDF2 antibodies. Results were presented as mean ± SD of three independent experiments. *P < 0.05 or **P < 0.01 indicates a significant difference between the indicated groups. ns, not significant

When m^6^A of pre-mRNA is recognized by YTHDFs, either decay or translation of the mRNA is promoted, depending on the different YTHDFs involved. YTHDF3 serves as a hub to fine-tune the accessibility of RNA to YTHDF1 and YTHDF2 [15]. We therefore propose that YTHDF3 recognizes m^6^A modification of YAP pre-mRNA. YTHDF3 next was competitively bound by YTHDF1 or YTHDF2 passing the *YAP* pre-mRNA carrying the m^6^A modification to YTHDF3’s combinative partner. This may influence *YAP* expression in a reaction mediated by m^6^A modification of *YAP* pre-mRNA in the normal lung tissues and NSCLC tumor tissues (Fig. 3a). First, we observed that YTHDF1, YTHDF2, and YTHDF3 bind to *YAP* mRNA in A549 and H1299 cells (Fig. 3b). Additionally, increasing m^6^A modification by knocking down *ALKBH5* significantly enhanced the interaction between YTHDF1/2/3 and *YAP* pre-mRNA, suggesting that YTHDF1/2/3 binding to *YAP* pre-mRNA is mediated by m^6^A modification (Fig. 3c). We then explored whether YTHDF1/2 interaction with *YAP* mRNA was affected by YTHDF3. Interestingly, RNA pulldown assays revealed that YTHDF1 or YTHDF2 binding to *YAP* mRNA was increased in the presence of YTHDF3, and less YTHDF1 or YTHDF2 bound *YAP* mRNA in the absence of YTHDF3 in A549 and H1299 cells (Fig. 3d, Additional file 6: Fig. S5a). We next explored whether YTHDF1 and YTHDF2 competitively bound YTHDF3. We found that ALKBH5 protein was only detectable in the nuclear fractions, enabling m^6^A modification of pre-mRNA (Fig. 3e, f). By contrast, YTHDFs proteins were localized only in the cytoplasmic fractions, in which *YAP* mRNA was destroyed or translated (Fig. 3e, g). However, the nuclear and cytoplasmic distribution of YAP Mut1–3 were not affected compared to YAP WT in A549 cells (Additional file 6: Fig. S5b, c). In addition, the endogenous co-IP experiments indicated that YTHDF1, YTHDF2, and YTHDF3 reciprocally and physically interacted with each other (Fig. 3h). Importantly, in A549 and H1299 cells, upon YTHDF1 overexpression, the interaction between YTHDF2 and YTHDF3 was inhibited; by contrast, when YTHDF2 was overexpressed, the interaction between YTHDF1 and YTHDF3 was inhibited (Fig. 3i). These observations indicated that YTHDF1 and YTHDF2 competitively bind YTHDF3 in NSCLC cells.

To investigated whether the m^6^A modification of *YAP* pre-mRNA was involved in the competition between YTHDF3 and both YTHDF1/2. First, we constructed the A549 and H1299 cells with stable expression of pcDNA3.1-YAP-MS2–12X (YAP-MS2) to utilize the MS2 coat protein system for enriching YAP mRNA (Additional file 6: Fig. S5d). Co-immunoprecipitation with western blotting analysis revealed that YTHDF1, YTHDF2, and YTHDF3 recognized m^6^A modification on *YAP* mRNA in A549 and H1299 cells (Fig. 3j, Additional file 6: Fig. S5e). Further, the amount of YTHDF1 and YTHDF2 proteins interacting with *YAP* pre-mRNA was decreased in A549 and H1299 cells after knocking down of *YTHDF3* (Fig. 3k). Interestingly, increased m^6^A modification by shALKBH5 preferentially endow YTHDF1′s interaction with YAP pre-mRNA compared to YTHDF2 in A549 and H1299 cells (Fig. 3l). This indicated that the m^6^A on *YAP* pre-mRNA was first recognized by YTHDF3 and then YTHDF1 and YTHDF2 competitively bound YTHDF3 next YTHDF3 passes the *YAP* pre-mRNA carrying the m^6^A modification to YTHDF3’s combinative partner.

Finally, we explored whether the competitive relationship between YTHDF1/2 and YTHDF3 was dependent on the m^6^A modification regulated by ALKBH5. Firstly, the level of m^6^A modification on *YAP* pre-mRNA was unchanged in A549 and H1299 cells co-transfected with shALKBH5 and YTHDF3, shALKBH5 and YTHDF1/2, or shALKBH5 and YTHDF3&1/2, compared with the levels after the shALKBH5 only transfection (Fig. 3m). This indicated that YTHDFs did not increase the level of m^6^A modification of *YAP* pre-mRNA but only ALKBH5 with demethylase could regulate the m^6^A modification of *YAP* pre-mRNA. In addition, increasing the m^6^A modification level by shALKBH5 or decreasing the m^6^A modification level by ALKBH5 did not affect the interaction between YTHDF1/2 and YTHDF3, as revealed by Co-IP analysis (Fig. 3n, Additional file 6: Fig. S5f). These findings indicated that YTHDF1/2 competitively binds YTHDF3 independently of the m^6^A modification.

In summary, we found that m^6^A on *YAP* pre-mRNA was first recognized by YTHDF3, which then competitively bound YTHDF1 or YTHDF2 in an m^6^A-independent manner to regulate *YAP* expression. However, YTHDF1/2/3 binding to *YAP* pre-mRNA was mediated by the m^6^A modification.

### YTHDF2-facilitated decay of *YAP* mRNA is mediated by AGO2 system and regulated by m^6^A modification

**Fig. 4:**
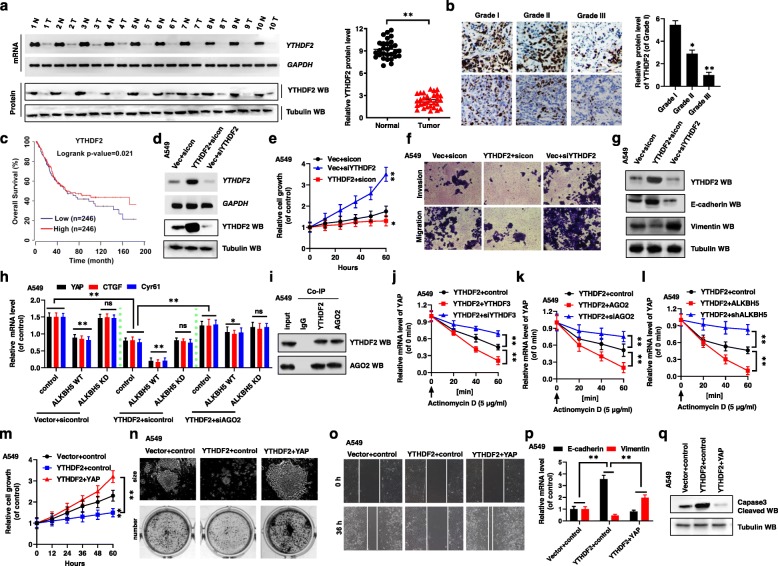
YTHDF2-facilitated decay of YAP mRNA is mediated by AGO2 system and regulated by m^6^A modification. (**a**) The mRNA and protein levels of YTHDF2 were analyzed in paired tumor cancer tissues (Tumor) and adjacent normal tissues (Normal) coming from BinZhou medical university hospital (left panel, *n* = 10; right panel, n = 30). (**b**) The expression of YTHDF2 was analyzed by IHC assay in the different grades of lung cancer tissues. (**c**) Low expression of YTHDF2 is associated with worse prognosis for NSCLC patients (*P = 0.021*). (**d-g**) A549 cells were transfected with indicated genes of YTHDF2. (**d**) The expressions of YTHDF2 were analyzed by RT-PCR and western blot assays. (**e**) The cellular growth was analyzed by CCK8 assay. (**f**) The invasion and migration growths were analyzed by transwell assay. (**g**) The expressions of E-cadherin and Vimentin were analyzed by western blot assay. (**h**) The mRNA levels of YAP, CTGF and Cyr61 were detected in A549 cells by qPCR assay. (**i**) Co-IPs performed using lysates collected from A549 cells with immunoprecipitation by either YTHDF2 or AGO2 antibodies, respectively. (**j-l**) The mRNA level of YAP was analyzed by qPCR in Actinomycin D treated A549 cells with transfected with indicated genes. (**m-q**) A549 cell were transfected with indicated genes of YTHDF2 and YAP. (**m**) The cellular growth was analyzed by CCK8 assay. (**n**) The size and number of colons were analyzed by colony formation assay. (**o**) The cellular migration growth was analyzed by scratch assay. (**p**) The expressions of E-cadherin and Vimentin were analyzed by qPCR assay. (**q**) The relative of cleaved Caspas-3 (Caspas-3-Cl) was analyzed by western blot. Results were presented as mean ± SD of three independent experiments. *P < 0.05 or **P < 0.01 indicates a significant difference between the indicated groups. ns, not significant

Recent studies have shown that, m^6^A mediates two different fates of mRNA, i.e., by promoting the decay or translation of mRNA. We therefore investigated whether and how the YTHDF proteins, which recognize the m^6^A modification, control NSCLC growth and migration by regulating *YAP* pre-mRNA status. As shown in Fig. 3a, YTHDF2 competitively bound YTHDF3 to promote *YAP* mRNA decay under control conditions. As indicated above, the mRNA and protein levels of YTHDF2 were higher in the NSCLC normal tissues than in their paired tumor tissues (Fig. 4a, Additional file 7: Fig. S6a, b). In addition, YTHDF2 protein levels were gradually decreasing with the increasing grade of NSCLC tumor (Fig. 4b). The correlation of individual the protein level of YTHDF2 with pathological grades of NSCLC patients was shown in Table S1 (Additional file 1: Table S1). Further, the mRNA and protein levels of YTHDF2 were lower in NSCLC cancer cells than in the control cells BEAS-2B (Additional file 7: Fig. S6c). Next, we used the publicly available datasets to determine the prognostic correlation between YTHDF2 expression and NSCLC patient survival [27]. The results show that the OS of patients with low YTHDF2 expression (*n* = 246) was shorter than that of patients with high YTHDF2 expression (n = 246) (*P = 0.021*) (Fig. 4c). To investigate the function of YTHDF2 in NSCLC cells, we knocked down *YTHDF2* using siYTHDF2–1 and siYTHDF2–2 (knocking down *YTHDF2* using siYTHDF2–1 was more effective than siYTHDF2–2, so siYTHDF2–1 was used to carry out then subsequent experiments) or overexpressed the protein by transfecting A549 and H1299 cells with a plasmid encoding Flag-YTHDF2 (Fig. 4d, Additional file 7: Fig. S6d, e). Knocking down of *YTHDF2* increased, while *YTHDF2* overexpression decreased, the cellular growth and viability of A549 and H1299 cells, as determined by the CCK8 assay (Fig. 4e, Additional file 7: Fig. S6f), determining cleaved caspase 3 levels (Additional file 7: Fig. S6g) and Ki67 staining (Additional file 7: Fig. S6h). Similar effects on cell migration and invasion growth were obtained with A549 and H1299 cells, as determined by transwell and scratch assays (Fig. 4f, Additional file 7: Fig. S6i, j). Further, YTHDF2 overexpression in A549 and H1299 cells decreased mRNA and protein levels of Vimentin and increased those of E-cadherin, whereas the opposite was observed upon knocking down of *YTHDF2* (Fig. 4g, Additional file 7: Fig. S6k). Furthermore, spearman rank correlation analysis of data in TCGA database revealed positive correlation between YTHDF2 and E-cadherin levels and negative correlation between YTHDF2 and vimentin levels (Additional file 7: Fig. S6l). These observations indicated that overexpression of YTHDF2 inhibits cellular proliferation, invasion, migration, and EMT in NSCLC cells.

Next, we explore the mechanism whereby YTHDF2 promoted to decay *YAP* mRNA. First, we observed that *YTHDF2* overexpression further reduced ALKBH5-mediated decrease of *YAP* mRNA levels, while cell co-transfection with siAGO2 reduced this effect (Fig. 4h, Additional file 8: Fig. S7a). Importantly, endogenous co-IP experiments revealed that YTHDF2 bound to argonaute RISC catalytic component 2 (AGO2) (Fig. 4i). This indicated that after YTHDF2 binding to YTHDF3, which binds *YAP* pre-mRNA modified by m^6^A, YTHDF2 presented *YAP* mRNA to AGO2, followed by AGO2 recruitment of other molecules, including some microRNAs, to form RISC system to facilitate decay of *YAP* mRNA. To accurately evaluate the decay of *YAP* mRNA associated with YTHDF2, we analyzed mRNA degradation by qPCR in actinomycin D (ActD)-treated A549 and H1299 cells. We observed that the *YAP* mRNA was decreased with increasing concentrations of ActD, while *YAP* mRNA levels increased or decreased in ActD-treated A549 and H1299 cells after knock down or overexpression of *YTHDF2*, respectively, compared with the control vectors (Additional file 8: Fig. S7b). In addition, with increasing ActD-treatment time, the decay of *YAP* mRNA was increased or decreased in A549 and H1299 cells transfected with *YTHDF2* or siYTHDF2, respectively, compared with the control vectors (Additional file 8: Fig. S7c, d). Further, the *YAP* mRNA degradation was increased or decreased in ActD-treated A549 and H1299 cells co-transfected with *YTHDF2* and *YTHDF3* or YTHDF2 and siYTHDF3, respectively, compared with the control (Fig. 4j, Additional file 8: Fig. S7e). The mRNA levels of *YAP*, *CTGF*, and *Cyr61* also showed these trends in the transfected cells (Additional file 8: Fig. S7f). Similarly, the *YAP* mRNA degradation was increased or decreased in ActD-treated A549 and H1299 cells co-transfected with *YTHDF2* and AGO2 or *YTHDF2* and siAGO2, respectively, compared with the control (Fig. 4k). Furthermore, YTHDF2-associated *YAP* mRNA degradation was increased in cells with decreased m^6^A status regulated by ALKBH5 but decreased in cells with increased m^6^A status regulated by shALKBH5 in NSCLC cells (Fig. 4l). Finally, the m^6^A-mediated *YAP* mRNA decay was increased upon co-transfection with *YTHDF2* and *AGO2* but decreased upon co-transfection with *YTHDF2* and *siAGO2*, compared with the control, in ActD-treated A549 and H1299 cells (Additional file 8: Fig. S7g). These observations indicated that YTHDF2-mediated *YAP* mRNA decay is regulated by m^6^A modification via the AGO2 system.

Next, we explored whether YTHDF2 inhibited the cellular growth, invasion, and EMT by regulating YAP (Additional file 8: Fig. S7h, i). Indeed, overexpression of YAP reversed the YTHDF2-associated inhibition of cellular growth and viability (Fig. 4m, Additional file 8: Fig. S7j), Ki67-positive cell staining (Additional file 8: Fig. S7k), clone formation (Fig. 4n, Additional file 8: Fig. S7l), migration (Fig. 4o, Additional file 8: Fig. S7m), invasion (Additional file 8: Fig. S7n), EMT (Fig. 4p, Additional file 8: Fig. S7o), and YTHDF2-associated promotion of apoptosis (Fig. 4q, Additional file 8: Fig. S7p) in A549 and H1299 cells. These observations indicated that *YAP* mRNA decay facilitated by YTHDF2 is mediated by the AGO2 system and regulated by m^6^A modification, inhibiting cellular growth, invasion, and EMT of NSCLC cells.

### YTHDF1-promoted *YAP* mRNA translation is regulated by m^6^A modification and interaction with eIF3a

**Fig. 5:**
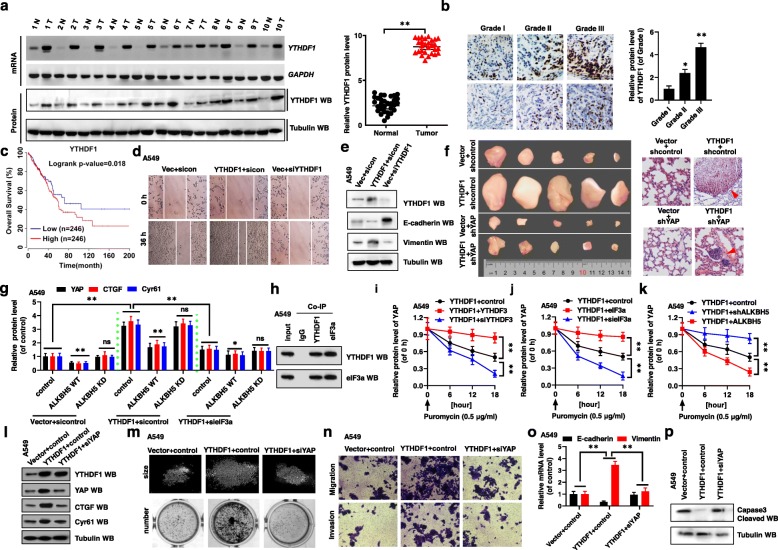
YTHDF1-promoted YAP mRNA translation is regulated by m^6^A modification and interaction with eIF3a. (**a**) The mRNA and protein levels of YTHDF1 were analyzed by RT-PCR and western blot assays in paired fresh NSCLC tumor cancer tissues (Tumor) and matched adjacent normal tissues (Normal) (left panel, n = 10; right panel, n = 30). (**b**) The expression of YTHDF1 was analyzed by IHC assay in the different grades of lung cancer tissues. (**c**) High expression of YTHDF1 is associated with worse prognosis for NSCLC patients (*P = 0.018*). (**d, e**) A549 cells were transfected with indicated genes of YTHDF1. (**d**) The migration growth was analyzed by scratch assay. (**e**) The expressions of E-cadherin and Vimentin were analyzed by western blot assay. (**f**) The xenografted tumors (left panel) and the metastasizing lung tumors (right panel) originated from A549 cells with stable expression of indicated genes constructed by subcutaneous injection. (**g)** The protein levels of YAP, CTGF and Cyr61 were analyzed in A549 cells determined by WB assay. (**h**) Co-IPs performed using lysates collected from A549 cells with immunoprecipitation by either YTHDF1 or eIF3a antibodies. (**i-k**) The protein level of YAP was analyzed by ELISA assay in puromycin treated A549 cells with transfection with indicated genes. (**l-p**) A549 cell were transfected with indicated genes of YTHDF1 and YAP. (**l**) The protein levels of YTHDF1, YAP, CTGF and Cyr61 were analyzed by western blot assay. (**m**) The size and number of colons were analyzed by colony formation assay. (**n**) The cellular invasion and migration growth were analyzed by transwell assay. (**o**) The expressions of E-cadherin and Vimentin were analyzed by qPCR assay. (**p**) The relative of cleaved Caspas-3 was analyzed by western blot assay. Results were presented as mean ± SD of three independent experiments. *P < 0.05 or **P < 0.01 indicates a significant difference between the indicated groups. ns, not significant

We next explored the mechanism whereby YTHDF1 promoted tumor growth and metastasis by regulating YAP expression in NSCLC. First, we observed that the expressions of YTHDF1 were higher in tumor tissues than in normal tissues (Fig. 5a, Additional file 9: Fig. S8a, b). IHC analysis revealed that YTHDF1 protein levels were gradually increased with increasing grade of tumor species in NSCLC (Fig. 5b). The correlation of individual the protein level of YTHDF1 with pathological grades of NSCLC patients was shown in Table S1 (Additional file 1: Table S1). In addition, the expressions of YTHDF1 were higher in the NSCLC tumor cell lines than that in the control cells BEAS-2B (Additional file 9: Fig. S8c). We also used the publicly available datasets to determine the prognostic correlation between YTHDF1 expression and NSCLC patient survival [27]. The results show that the OS of patients with high YTHDF1 expression (*n* = 246) was shorter than that of patients with low YTHDF1 expression (n = 246) (*P* = 0.018) (Fig. 5c). Next, to investigate the function of YTHDF1 in the occurrence and development of NSCLC, we overexpressed YTHDF1 by transfecting A549 and H1299 cells with pcDNA 3.1-YTHDF1, and knocking down of *YTHDF1* by siYTHDF1–1 and siYTHDF1–2 (knocking down *YTHDF1* using siYTHDF1–2 was more effective than siYTHDF1–1, so siYTHDF1–2 was used to carry out then subsequent experiments) in A549 and H1299 cells (Additional file 9: Fig. S8d, e). Overexpression of *YTHDF1* promoted cellular viability (Additional file 9: Fig. S8f), growth (Additional file 9: Fig. S8g, h), migration (Fig. 5d, Additional file 9: Fig. S8i), invasion (Additional file 9: Fig. S8j), and EMT (Fig. 5e, Additional file 9: Fig. S8k), but inhibited apoptosis (Additional file 9: Fig. S8l) of A549 and H1299 cells. The opposite results were seen in A549 and H1299 cells after *YTHDF1* was knocked down. Further, TCGA database analysis indicated that YTHDF1 expression was negatively correlated with E-cadherin expression but positively correlated with vimentin expression (Additional file 9: Fig. S8m) in the NSCLC. Furthermore, YTHDF1 overexpression promoted xenograft tumor growth in vivo (Fig. 5f, left panel, Additional file 9: Fig. S8n-p). The similar xenograft tumor metastatic ability and mice survival were obtained in the same mouse group (Fig. 5f, right panel, Additional file 9: Fig. S8q). These observations suggested that YTHDF1 promotes tumor growth and metastasis in NSCLC.

Next, we explored the mechanism whereby YTHDF1 promoted *YAP* mRNA translation (Fig. 3a). Firstly, we observed that *YTHDF1* overexpression increased the protein levels of YAP, CTGF, and Cyr61. However, overexpression of *ALKBH5* WT but not *ALKBH5* KD reversed the enhanced expression of these genes regulated by YTHDF1 (Fig. 5g, Additional file 10: Fig. S9a). In addition, the protein levels of YAP, CTGF, and Cyr61 were reduced in A549 and H1299 cell co-transfected with *YTHDF1* and sieIF3a (eIF3a is a eukaryotic translation initiation factor 3) compared with cells only transfected with *YTHDF1* (Fig. 5g, Additional file 10: Fig. S9a). The endogenous co-IP experiments showed that YTHDF1 interacted to eIF3a (Fig. 5h). These data indicated that once YTHDF1 binds to YTHDF3, which binds *YAP* mRNA containing m^6^A, YTHDF1 presents *YAP* mRNA to eIF3a-contained translation initiation complex to promote *YAP* mRNA translation. We then treated A549 and H1299 cells with puromycin dihydrochloride (protein synthesis inhibitor) to evaluate YTHDF1-associated *YAP* mRNA translation by quantitative ELISA assay. Results showed the *YAP* mRNA translation was inhibited with increasing concentration of puromycin, while YAP protein levels increased or decreased in puromycin-treated A549 and H1299 cells upon overexpression or knockdown of *YTHDF1*, respectively, compared with the control vectors (Additional file 10: Fig. S9b). In addition, with increasing puromycin-treatment time, *YAP* mRNA translation increased or decreased in A549 and H1299 cells transfected with *YTHDF1* or siYTHDF1, respectively, compared with the control vectors (Additional file 10: Fig. S9c). Furthermore, *YAP* mRNA translation increased or decreased in puromycin-treated A549 and H1299 cells co-transfected with *YTHDF1*, and *YTHDF3* or siYTHDF3, respectively, compared with the control (Fig. 5i, Additional file 10: Fig. S9d). The same results were observed for the protein levels of YAP, CTGF, and Cyr61 in these transfected cells (Additional file 10: Fig. S9e). Similar, *YAP* mRNA translation increased or decreased in puromycin-treated A549 and H1299 cells co-transfected with *YTHDF1*, and *eIF3a* or sieIF3a, respectively, compared with the control (Fig. 5j, Additional file 10: Fig. S9f). Further, YTHDF1-associated *YAP* mRNA translation was increased in cells with increased m^6^A status regulated by shALKBH5 but decreased in cells with reduced m^6^A status regulated by ALKBH5 (Fig. 5k, Additional file 10: Fig. S9g). Finally, m^6^A-mediated *YAP* mRNA translation increased upon co-transfection into puromycin-treated A549 and H1299 cells with *YTHDF1* and *eIF3a*, but decreased after co-transfection with YTHDF1 and *sieIF3a*, compared with the control (Additional file 10: Fig. S9h). These findings suggested that YTHDF1/eIF3a-mediated *YAP* mRNA translation is controlled by m^6^A modification.

Next, we investigated whether YTHDF1 increased NSCLC tumor growth and metastasis by regulating YAP (Fig. 5l, Additional file 10: Fig. S9i). Knockdown of *YAP* inhibited the YTHDF1-mediated improvement of cellular growth (Additional file 10: Fig. S9j), viability (Additional file 10: Fig. S9k), Ki67-positive cell staining (Additional file 10: Fig. S9l), clone formation (Fig. 5m, Additional file 10: Fig. S9m), migration (Additional file 10: Fig. S9n), invasion (Fig. 5n, Additional file 10: Fig. S9o), EMT (Fig. 5o, Additional file 10: Fig. S9p), and YTHDF1-associated inhibition of apoptosis (Fig. 5p, Additional file 10: Fig. S9q) in A549 and H1299 cells. Moreover, stable knockdown of YAP inhibited xenograft tumor growth mediated by YTHDF1 overexpression in vivo (Fig. 5f, left panel, Additional file 9: Fig. S8o, p). The similar xenograft tumor metastatic ability and mice survival were obtained in the mouse group by stable knockdown of YAP (Fig. 5f, right panel, Additional file 9: Fig. S8q). This indicated that YTHDF1 promotes *YAP* mRNA translation, which enhances cellular growth, invasion, and EMT of NSCLC cells in vitro and in vivo.

### ALKBH5 decreases YAP activity by regulating miR-107/LATS2 in an HuR-dependent manner

**Fig. 6:**
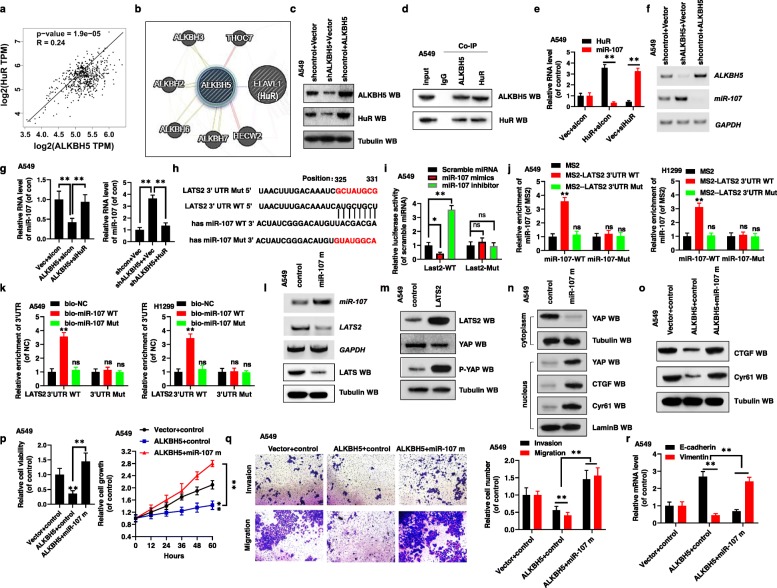
ALKBH5 decreases YAP activity by regulating miR-107/LATS2 in an HuR-dependent manner. (**a**) The positive correlation between ALKBH5 and HuR analyzed from TCGA database. (**b**) Bioinformatics prediction the interaction between ALKBH5 and HuR (https://thebiogrid.org/). (**c**) The protein levels of ALKBH5 and HuR was analyzed by western blot assay. (**d**) Co-IPs performed using lysates collected from A549 cells with immunoprecipitation by either ALKBH5 or HuR antibodies. (**e-g**) A549 cells were transfected into indicated genes. The RNA levels of HuR, ALKBH5 and miR-107 were analyzed by qPCR and RT-PCR. (**h**) Putative miR-107 binding sites in the 3′UTR sequences of LAST2. (**i**) The luciferase reporter activity of the wild-type and mutated LATS2 was detected in A549 cells. (**j**) MS2-RIP followed by qPCR to measure miR-107 associated with 3′UTR of LATS2 in A549 and H1299 cells. (**k**) qPCR analyzed the RNA levels of miR-107 and LATS2 3’UTR in the products of A549 and H1299 cells with transfection with indicated genes by pulldown with biotin. (**l, m**) A549 cells were transfected into indicated genes. The RNA and protein levels of miR-107, LAST2, YAP and p-YAP were analyzed by RT-PCR and western blot assays. (**n**) Subcellular localization of YAP, CTGF and Cyr61 were examined in A549 cells determined by western blot assay. (**o-r**) A549 cells were transfected with indicated genes of ALKBH5 and miR-107 mimics (miR-107 m). (**o**) The protein levels of CTGF and Cyr61 were analyzed by western blot assay. (**p**) The cellular viability and growth were analyzed by CCK8 assay. (**q**) The cellular invasion and migration growths were analyzed by transwell. (**r**) The expressions of E-cadherin and Vimentin were analyzed by qPCR assay. Results were presented as mean ± SD of three independent experiments. **P < 0.01 indicates a significant difference between the indicated groups. ns, not significant

ALKBH5 regulates the expression of Human antigen R (HuR), which is a posttranscriptional regulator of gene expression, played a key role in stabilizing multiple mRNAs in cellular biology [12, 13]. Analysis of information in the TCGA database revealed a positive correlation between ALKBH5 and HuR mRNA levels in NSCLC tissues (Fig. 6a). Further, bioinformatics analysis suggested that ALKBH5 interacts with HuR (Fig. 6b). While overexpression of *ALKBH5* caused increase in HuR expression, silencing of *ALKBH5* resulted in a significantly decreased expression of HuR in A549 and H1299 cells (Fig. 6c). Interestingly, the expression of HuR was unchanged in A549 and H1299 cells transfected with *ALKBH5* KD, compared with the control (Additional file 11: Fig. S10a). We then confirmed the physical interaction between HuR and ALKBH5 by endogenous Co-IP assay in A549 cells (Fig. 6d). These findings indicated that ALKBH5 might be involved in regulating the expression of HuR.

Recent studies have shown that miR-107 regulates proliferation and invasion of gastric adenocarcinoma cells by regulation of LATS2 [31]. However, the underlying mechanism of miR-107 especially in association with tumor growth and metastasis in NSCLC has not been explored. Coupled with that ALKBH5 was involved in regulating the expression of HuR meantime HuR played a key role in stability of multiple RNAs, we assume that ALKBH5 regulate the miR-107 level in an HuR-dependent manner in NSCLC. Importantly, qPCR analysis revealed that HuR repressed the miR-107 expression by regulating its processing (Fig. 6e, Additional file 11: Fig. S10b). It remained to be elucidated whether ALKBH5 regulates miR-107 expression in an HuR-dependent manner. As anticipated, ectopic expression of *ALKBH5* inhibited the expression of miR-107 (Fig. 6f); however, this regulation was inhibited by the silencing of HuR (Fig. 6g, Additional file 11: Fig. S10c). Furthermore, silencing *ALKBH5* elevated the expression of miR-107, which, in turn, was inhibited by the ectopic expression of HuR (Fig. 6g, Additional file 11: Fig. S10c). These observations indicated that ALKBH5 decreases the miR-107 levels in an HuR-dependent manner.

Since ALKBH5 decreases the miR-107 level, we would seek the specific target gene for miR-107 and explore whether ALKBH5 control the activity of YAP via regulation of miR-107 in NSCLC. Interestingly, according to miRbase, miRanda, and TargetScan database analysis, *LATS2*, which inhibits the proliferation and migration of tumor via regulating the activity of YAP, is a direct target of miR-107 (Fig. 6h). Dual luciferase reporter assay confirmed that LATS2 is a direct target of miR-107 (Fig. 6i, Additional file 11: Fig. S10d). MS2 binding system (Fig. 6j) and RNA pulldown approach combined with qPCR quantification involving a biotin-labeled probe (Fig. 6k, Additional file 11: Fig. S10e) revealed that miR-107 directly binds to 3′UTR of *LATS2*. In addition, miR-107 mimics (miR-107 m) inhibited, while miR-107 inhibitors (miR-107 i) elevated, the expression of *LATS2* (Fig. 6l, Additional file 11: Fig. S10f, g). In addition, TCGA database analysis revealed a positive correlation between ALKBH5/HuR, which regulate the miR-107 level, and LAST2 in NSCLC tumors (Additional file 11: Fig. S10h). These findings indicated that miR-107 regulation by ALKBH5 reduces the expression of *LAST2* in an HuR-dependent manner.

By using western blot and immunofluorescence staining, we showed that LATS2 regulates the activity of YAP by promoting its phosphorylation (Fig. 6m, Additional file 11: Fig. S10i). We also observed that miR-107 mimics reduced the phosphorylation of YAP and increased the nuclear translocation of YAP, increasing YAP activity (as determined by analyzing the expression of CTGF and Cyr61) (Fig. 6n, Additional file 11: Fig. S10j). This suggested that miR-107 increased YAP activity by reducing LAST2 levels in NSCLC. Finally, we explored whether ALKBH5 decreased the activity of YAP via miR-107. Indeed, protein levels of CTGF and Cyr61 were reversed in the A549 and H1299 cells co-transfected with *ALKBH5* and miR-107 mimics compared with cells transfected with *ALKBH5* alone (Fig. 6o, Additional file 11: Fig. S10k). Furthermore, co-transfection of A549 and H1299 cells with *ALKBH5* and miR-107 mimics reversed the ALKBH5-associated inhibition of cell viability and growth (Fig. 6p, Additional file 11: Fig. S10l), invasion and migration (Fig. 6q, Additional file 11: Fig. S10m), and EMT (Fig. 6r, Additional file 11: Fig. S10n). Furthermore, co-transfection of A549 and H1299 cells with *ALKBH5* and siLAST2 exerted a similar effect as that of the co-transfection of *ALKBH5* and miR-107 on the expression of CTGF and Cyr61 (Additional file 11: Fig. S10o), cell viability (Additional file 11: Fig. S10p), invasion and migration (Additional file 11: Fig. S10q), and EMT (Additional file 11: Fig. S10r).

Collectively, the above findings indicated that ALKBH5 decreases the activity of YAP by regulating miR-107/LATS2 axis in an HuR-dependent manner.

### ALKBH5 inhibits tumor growth and metastasis by reducing the expression and activity of YAP in YTHDF1/2- and miR-107–dependent manner in vivo

**Fig. 7:**
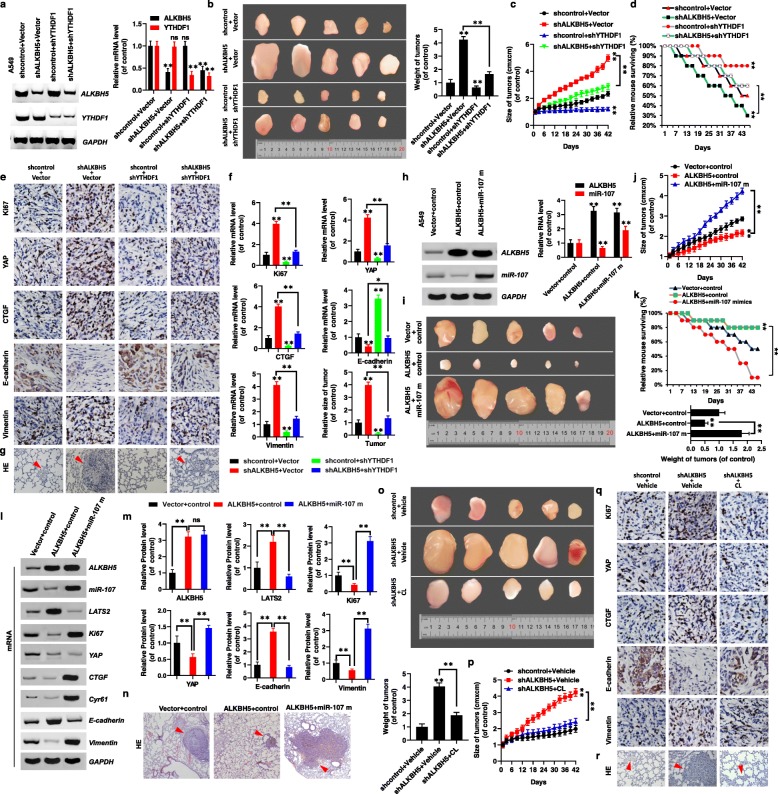
ALKBH5 inhibits tumor growth and metastasis by reducing the expression and activity of YAP in YTHDF1/2 and miR-107-dependent manner in vivo. (**a**) The expressions of ALKBH5 and YTHDF1 were analyzed by RT-PCR and qPCR assays. (**b, c**) Xenografted A549 cell tumors with stable expression of indicated genes in mice (**b**) and the dimensions measured at regular intervals (**c**). (**d**) The overall survival (OS) curves of the mice from transfected of A549 cells with stable expression of indicated genes. (**e, f**) The protein (**e**) and mRNA (**f**) levels of Ki67, YAP, CTGF, E-cadherin and Vimentin were detected in xenografted A549 cell tumors with stable expression of indicated genes determined by immunohistochemical staining (*n* = 5) and qPCR assays. (**g**) Representative H&E stained microscopic images of the mice metastatic lung tumors. (**h**) The RNA levels of ALKBH5 and miR-107 were analyzed by RT-PCR and qPCR in the A549 cells with stable expression of indicated genes. (**i, j**) Xenografted A549 cell tumors with stable expression of indicated genes in mice (**i**) and the dimensions measured at regular intervals (**j**). (**k**) The OS curves of the mice with indicated A549 cells. (**l, m**) The mRNA (**l**) and protein (**m**) levels of indicated genes were detected in relevant xenografted A549 cell tumors determined by RT-PCR and western blot assays. (**n**) Representative H&E stained microscopic images of the mice metastatic lung tumors. (**o, p**) Xenografted A549 cell tumors with stable expression of indicated genes (**o**) and the dimensions measured at regular intervals in mice with treatment of vehicle or cycloleucine (CL) (**p**). (**q**) The protein levels of indicated genes were detected in relevant xenografted A549 cell tumors determined by IHC assay. (**r**) Representative H&E stained microscopic images of the mice metastatic lung tumors. Results were presented as mean ± SD of three independent experiments. **P < 0.01 indicates a significant difference between the indicated groups

Based on the above findings regarding the roles of ALKBH5, YTHDF1 and miR-107 in NSCLC tumor growth and metastasis, we generated A549 cell lines stably co-expressing *shALKBH5* with an empty vector (*shALKBH5*^*Vector*^), *shYTHDF1* with shcontrol (*shYTHDF1*^*shcontrol*^), *shALKBH5* with *shYTHDF1* (*shALKBH5*^*shYTHDF1*^) and a control stable cell line, *shcontrol*^Vector^. We then used these cells to generate a mouse xenograft model. We first analyzed the expression levels of *ALKBH5* and *YTHDF1* by RT-PCR and qPCR to validate the generated cell lines (Fig. 7a). Approximately 2 weeks after subcutaneous implantation of these cells into mice, larger tumors (Fig. 7b) and faster tumor growth (Fig. 7c) were observed in *shALKBH5*^*Vector*^ group, and opposite in the *shYTHDF1*^*shcontrol*^ group compared with the tumor weight and growth in the *shcontrol*^Vector^ group. In addition, *shALKBH5*^*shYTHDF1*^ decreased the tumor weight and growth compared to the *shALKBH5*^*Vector*^ group (Fig. 7b, c). Additionally, increased survival was observed in the *shYTHDF1*^*shcontrol*^ group, and decreased survival in the *shALKBH5*^*Vector*^ group, compared with the survival in the *shcontrol*^Vector^ group (Fig. 7d). The survival was increased in *shALKBH5*^*shYTHDF1*^ group compared to the *shALKBH5*^*Vector*^ group (Fig. 7d). Moreover, quantitative IHC analysis (*n* = 5) (Fig. 7e) and qPCR assays (Fig. 7f) of Ki67, YAP, CTGF, Cyr61, and Vimentin expression in the xenografts revealed higher levels of their expressions in the *shALKBH5*^*Vector*^ group than in the *shcontrol*^Vector^ group, with lower expression observed for E-cadherin and cleaved caspase 3 levels (Fig. 7e, f, Additional file 12: Fig. S11a, b). The opposite was observed in the *shYTHDF1*^*shcontrol*^ group compared with their expressions in the *shcontrol*^Vector^ group (Fig. 7e, f, Additional file 12: Fig. S11a, b). Further, *shALKBH5*^*shYTHDF1*^ reversed these expressions within the *shALKBH5*^*Vector*^ group (Fig. 7e, f, Additional file 12: Fig. S11a, b). Furthermore, significantly more and larger lung cancer metastatic lesions were observed in the *shALKBH5*^*Vector*^ group, and fewer and smaller metastatic lesions in the *shYTHDF1*^*shcontrol*^ group, compared with the *shcontrol*^Vector^ (Fig. 7g). Typically, *shALKBH5*^*shYTHDF1*^ decreased the tumor metastasis compared with the *shALKBH5*^*Vector*^ group. These observations indicated that ALKBH5 controls tumor growth and metastasis by regulating the expression of YAP in an YTHDF1-dependent manner in vivo.

Next, we generated an A549 cell line stably co-expressing *ALKBH5* with a control vector (*ALKBH5*^*control*^) or miR-107 mimics (*ALKBH5*^*miR-107 m*^), and a control stable cell line, Vector^control^, to explore whether miR-107 was involved in the ALKBH5-mediated tumor growth and metastasis. The expression levels of *ALKBH5* and miR-107 were analyzed by RT-PCR and qPCR to validate the generated cell lines (Fig. 7h). Larger tumors (Fig. 7i) and faster tumor growth (Fig. 7j) were observed in *ALKBH5*^*miR-107 m*^ group than in the *ALKBH5*^*control*^ group. In addition, the survival in the *ALKBH5*^*miR-107 m*^ group was decreased compared with that in the *ALKBH5*^*control*^ group (Fig. 7k). The expression of Ki67, CTGF, Cyr61, and Vimentin was increased but that of E-cadherin and cleaved caspase 3 was decreased in the *ALKBH5*^*miR-107 m*^ group compared with the *ALKBH5*^*control*^ group, as determined by RT-PCR and IHC analysis (n = 5) (Fig. 7l, m, Additional file 12: Fig. S11c). Further, significantly more and larger lung cancer metastatic lesions were observed in the *ALKBH5*^*miR-107 m*^ group than in the *ALKBH5*^*control*^ group (Fig. 7n). These observations indicated that ALKBH5 inhibits tumor growth and metastasis by reducing YAP activity in an miR-107–dependent manner in vivo.

To further explore whether inhibition of m^6^A decreased the tumor growth and metastasis, the m^6^A inhibitor, cycloleucine (CL) [32] which could negatively regulate m^6^A levels by decreasing the S-adenosyl-methionine concentration, was used in this study. Our results showed that cycloleucine significantly decreased the level of YAP m^6^A modification induced by shALKBH5, while not affecting the mRNA expression of ALKBH5 in A549 and H1299 cells (Additional file 12: Fig. S11d-f). In addition, treatment with cycloleucine significantly decreased the cell growth (Additional file 12: Fig. S11g), migration (Additional file 12: Fig. S11h) and EMT (Additional file12: Fig. S11i) in ALKBH5 transfected A549 and H1299 cells compared with treatment with Vehicle. Moreover, we generated A549 cell lines stably co-expressing *shALKBH5* with an empty vector (*shALKBH5*^*Vector*^) and a control stable cell line, *shcontrol*^Vector^, which were treated with cycloleucine (25 mg/kg twice weekly) and vehicle, respectively. Our data showed that the weight and growth of tumors were significantly decreased in the *shALKBH5*^*Vector*^ group with treatment of cycloleucine compared to the vehicle treatment (Fig. 7). Furthermore, quantitative IHC analysis (n = 5) (Fig. 7q, Additional file 12: Fig. S11j) and qPCR (Additional file12: Fig. S11k) assays of Ki67, YAP, CTGF, Cyr61, and Vimentin expression in the xenografts revealed lower levels of their expressions in the cycloleucine-treated than the vehicle--treated *shALKBH5*^*Vector*^ group, with higher expression observed for E-cadherin and cleaved caspase 3 levels (Fig. 7q, Additional file 12: Fig. S11j, k). These finding showed that a pharmacological inhibitor of m^6^A could inhibited tumor growth and metastasis.

## Discussion

Lung cancer is a common malignant tumor, classified into small cell lung cancer (SCLC) and NSCLC. NSCLC accounts for approximately 85% of lung cancer cases [33]. In recent years, although the treatment of NSCLC has improved, the 5-year survival rate has not improved significantly. After the treatment of early NSCLC, approximately 20% of patients have distant metastasis [34]. The specific mechanism of NSCLC tumor growth and distant metastasis remains unclear. In the current study, we clarified the molecular mechanism of m^6^A modification and function of *YAP*, regulated by ALKBH5 and YTHDFs proteins, in the regulation of NSCLC tumor growth and metastasis. The presented findings indicate that m^6^A modification of *YAP* is a novel target for a potential NSCLC therapy.

m^6^A is a conservative post-transcriptional modification, accounting for more than 60% of all RNA modifications. Proteins responsible for the addition, removal, and recognition of m^6^A can be divided into three categories: writers, erasers, and readers, respectively. The dynamic and reversible m^6^A modification is regulated by methylase and demethylase. The m^6^A methyltransferase complex, the “writer”, is primarily composed of methyltransferase-like 3 (mettl3) and 14 (mettl14), and Wilms tumor 1-binding protein (wtap) [14]. Demethylase, the “eraser”, is mainly composed of FTO and ALKBH5. ALKBH5 co-localizes with nuclear speckles to regulate the assembly/modification of mRNA processing factors, demethylate m^6^A mRNA, and modulate mRNA export and stability [35]. Therefore, loss of function of ALKBH5 leads to the disorder of many biological functions, for example, in *alkbh5*-deficient cells, because of accelerated nuclear RNA output, the cytoplasmic RNA levels increase significantly, the overall RNA stability decreases, and spermatocytes undergo apoptosis [7]. In addition, *alkbh5* gene knockout increases exon hopping, resulting in rapid degradation of transcripts with abnormal splicing [36]. We here showed that ectopic expression of *ALKBH5* inhibited NSCLC cell proliferation, invasion, migration, and EMT (Fig. 1). Importantly, ALKBH5 decreased the m^6^A level of *YAP* and inhibited *YAP* expression in NSCLC in a m^6^A dependent manner (Fig. 2). Furthermore, a pharmacological inhibitor of m^6^A, cycloleucine, could inhibited tumor growth and metastasis (Fig. 7), which indicated m^6^A modification plays an important role in the development and progression of human tumors.

The m^6^A binding proteins (“readers”) are primarily the YTH domain protein family (YTHDF1/2/3), nuclear heterogeneous protein HNRNP family, and IGF2BP protein family [15]. In the current study, we showed that the m^6^A modification of *YAP* pre-mRNA is first recognized by YTHDF3, which is followed by a competitive binding of YTHDF1 and YTHDF2 to YTHDF3, to decide the fate of *YAP* pre-mRNA, i.e., decay or translation (Fig. 3a). Specifically, recognition and binding of m^6^A mRNA sites by YTHDF3 and YTHDF2 led to mRNA transfer from a translatable pool to mRNA degradation site. After YTHDF2 binds YTHDF3, which carries *YAP* pre-mRNA containing m^6^A, YTHDF2 presents *YAP* mRNA to AGO2, and then AGO2 recruits other molecules to form RISC system to facilitate *YAP* mRNA decay, reducing YAP protein level. It has been recently shown that microRNAs, such as miR-195, miR-375, and others, decrease *YAP* mRNA levels by binding to *YAP* 3′UTR [37, 38]. Conversely, on the mRNA translation level, recognition and binding of m^6^A by YTHDF1 and YTHDF3 result in enhanced protein synthesis. As shown in the current study, following YTHDF1 binding of YTHDF3, which carries *YAP* pre-mRNA with m^6^A modification, YTHDF1 presents *YAP* mRNA to eIF3a-contained translation initiation complex to promote *YAP* mRNA translation, consistently with previous reports [39], leading to increased YAP protein levels. These observations account for regulation of YAP expression by m^6^A modification, i.e., by balancing the function of YTHDF1 and YTHDF2 via the YTHDF3 hub in NSCLC tumor and normal tissues. Specifically, the expression of YTHDF2 was higher in normal tissues than in tumor tissues (Fig. 4). This indicates that after YTHDF3 recognizes m^6^A modification on *YAP* mRNA, YTHDF2 was more likely to bind to YTHDF3, promoting *YAP* pre-mRNA decay, and thus maintaining normal development and growth of an organism. By contrast, the expression of YTHDF1 was higher in tumor tissues than in normal tissues (Fig. 5). This indicates that after YTHDF3 recognizes m^6^A modification on *YAP* mRNA, YTHDF1 was more likely to bind to YTHDF3, promoting *YAP* mRNA translation, and thus excessive cell growth and metastasis in NSCLC.

miRNAs interact with specific mRNA to degrade the corresponding target mRNA, thus inhibiting the translation of target mRNA and widely participating in the life processes of an organism, such as growth, development, differentiation, metabolism, and defense responses [40, 41]. Further, significant differences in the expression of various miRNAs in normal and tumor cells have been reported [23]. These miRNAs play a role similar to that of proto-oncogenes or tumor suppressor genes, by regulating different target genes, which are closely related to the occurrence, development, clinical treatment, and prognosis of many tumors [24]. Furthermore, HuR inhibits translation inhibition mediated by some miRNAs by directly binding and sequestering microRNA. Recently, HuR was shown to interact with such miRNAs as mir-16, mir-1192, and mir-29, to regulate the expression of *COX-2*, *HMGB1*, and *A20* genes, respectively [42–44]. In the case of miR-107, we here demonstrated that HuR binds to miR-107 as a miRNA sponge and blocks the translation inhibition of LATS2. Panneerdoss et al. (2018) showed that ALKBH5 increases the expression of HuR [13], in agreement with the findings of the current study. It remains unclear as to how ALKBH5 regulates HuR; however, we here showed that ALKBH5 KD does not regulate the HuR protein levels, indicating that m^6^A modification is possibly involved in the regulation of HuR. In other words, ALKBH5 enhances LATS2 expression by regulating the HuR/miR-107 axis. LATS2 phosphorylates YAP to decrease YAP activity and promote the interaction with 14–3-3 protein for YAP degradation. Collectively, ALKBH5 decreased the activity of YAP by regulating miR-107/LATS2 axis in an HuR-dependent manner (Fig. 6).

Most recent studies have shown that, the MST/Hippo signaling pathway is closely associated with the biological evolution and development, playing an important role in the regulation of cell proliferation, survival, cell signal transduction, organ generation, stem cell self-renewal, and organ volume [22]. Therefore, this signaling pathway has attracted increasing interest in the scientific community [45, 46]. Several key proteins in the MST/Hippo signaling pathway participate in tumor formation and apoptosis. The pathway contains multiple cell signal switches, and various signal molecules coordinate with each other to regulate cell proliferation and apoptosis [47]. The MST/Hippo signal pathway might be used to inhibit the growth of tumor cells, by regulating the Yap protein as a tumor suppressor. Yap activation is coordinated by the TEAD protein, and drugs may be used to inhibit the formation of the YAP–TEAD complex to block cell proliferation, which eventually leads to tumor apoptosis and death. Using this mechanism for drug research and development might provide valuable reference for the development of anti-tumor treatment. Specific drugs that block this signaling pathway, such as verteporfin, can inhibit cell proliferation by blocking the interaction between YAP and TEAD [48]. Therefore, in-depth study of the MST/Hippo signaling pathway could clarify the precise mechanism of tumor formation, further guiding and improving the clinical treatment, and providing a theoretical basis for the discovery of unknown tumor therapeutic targets.

Our study was not free of limitations: for example, the specific microRNA was involved in the AGO2-mediated YAP mRNA decay. Although we specified some microRNAs such as miR-195, miR-847-3p, et al.; a certain report indicated that YTHDF2 promotes cancer cell growth, which is contrary to our findings. One possible explanation is that when YTHDF2 interacts with tumor suppressor genes then promotes cell growth. While when YTHDF2 interacts with oncogenes then inhibits cell growth; unknown molecular mechanisms that higher expressions of YTHDF1 were in tumor tissues while higher expressions of YTHDF2 were in normal tissues of NSCLC; whether the regulatory mechanism mentioned above of ALKBH5 and YTHDFs applies to other genes or is limited to YAP need to be further explored. Therefore, these unresolved limitations need to be addressed in future studies.

## Conclusions

**Fig. 8:**
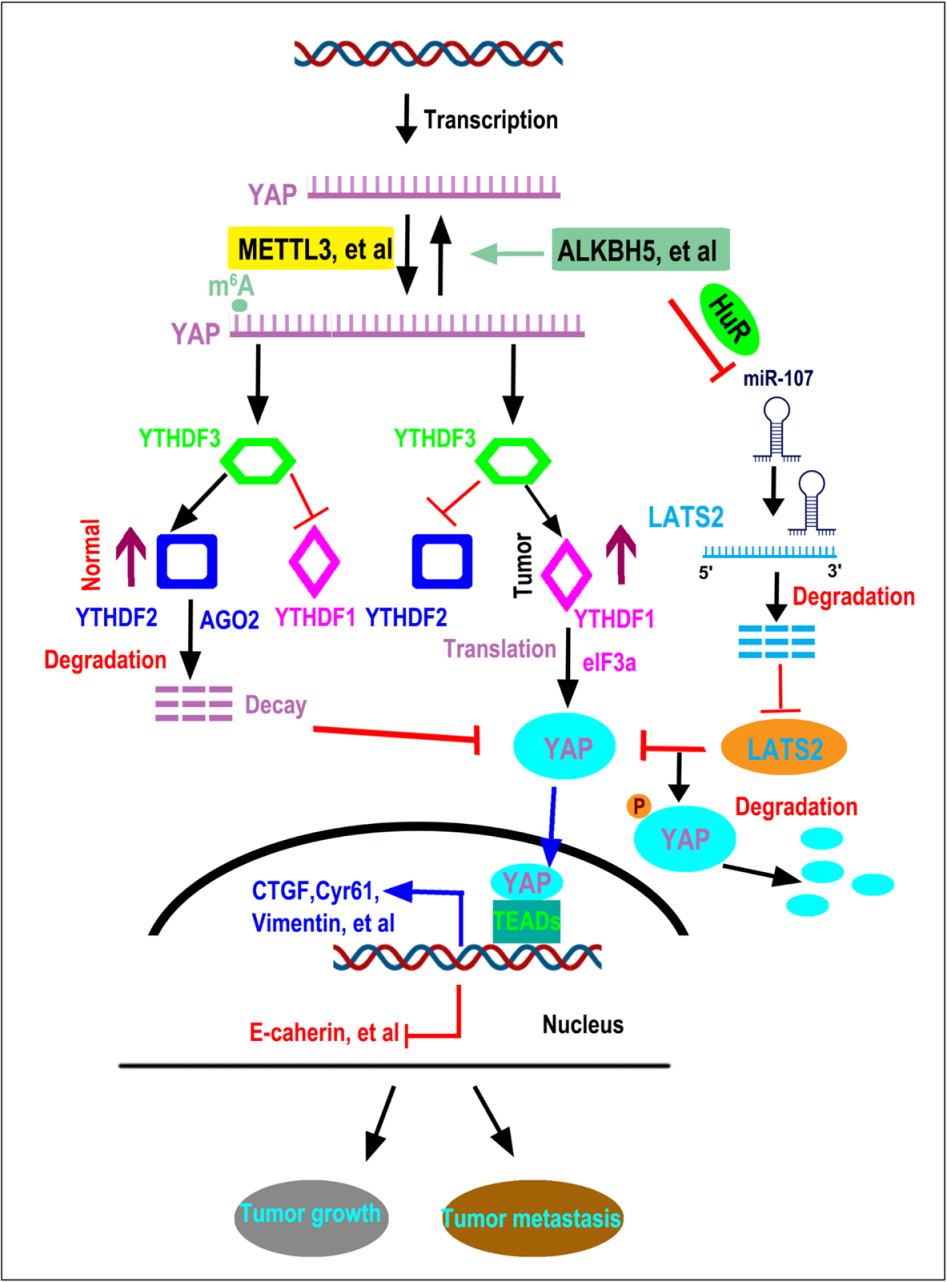
The diagram of ALKBH5 inhibits NSCLC growth and metastasis by regulating YAP expression and activity

We observed that YAP expression is negatively correlated with ALKBH5 expression, which plays an opposite role in the regulation of NSCLC tumor growth and metastasis. In addition, ALKBH5 decreased the m^6^A modification of *YAP* mRNA. The m^6^A of pre-mRNA was first recognized by YTHDF3, and then YTHDF1 and YTHDF2 competitively bound YTHDF3 to regulate *YAP* expression. Further, YTHDF2 facilitates *YAP* mRNA decay via the AGO2 system in normal tissue, while YTHDF1 promoted *YAP* mRNA translation by interacting with eIF3a in tumor tissue. Furthermore, ALKBH5 decreased the activity of YAP by regulating miR-107/LATS2 in an HuR-dependent manner. These functions result in ALKBH5 inhibition of tumor growth and metastasis by reducing the expression and activity of YAP in vivo. Therefore, m^6^A demethylase ALKBH5 inhibits tumor growth and metastasis by reducing YTHDFs-mediated *YAP* expression and inhibiting miR-107/LATS2–mediated YAP activity in NSCLC (Fig. 8). Thus, effective inhibition of *YAP* m^6^A modification could comprise a potential treatment strategy for lung cancer.

## Supplementary information

**Additional file 1 Table S1**. Correlation of ALKBH5, YAP, YTHDF1 and YTHDF2 with pathological grades of NSCLC patients.

**Additional file 2 Fig. S1**. Ectopic expression of YAP and ALKBH5 regulates cell proliferation.

**Additional file 3 Fig. S2.** Ectopic expression of YAP and ALKBH5 regulates cell migration, invasion and EMT.

**Additional file 4 Fig. S3**. ALKBH5 controls YAP expression by regulation m^6^A level in NSCLC.

**Additional file 5 Fig. S4.** ALKBH5 inhibits cell growth, migration and EMT by regulation of YAP.

**Additional file 6 Fig. S5.** YTHDF1 and YTHDF2 competitively interacts with YTHDF3.

**Additional file 7 Fig. S6.** YTHDF2 inhibits tumor growth and metastasis in NSCLC.

**Additional file 8 Fig. S7**. YTHDF2-facilitated decay of *YAP* mRNA is mediated by AGO2 system.

**Additional file 9 Fig. S8**. YTHDF1 promotes tumor growth and metastasis in NSCLC.

**Additional file 10 Fig. S9**. YTHDF1-promoted *YAP* mRNA translation is regulated by eIF3a.

**Additional file 11 Fig. S10**. ALKBH5 decreases YAP activity.

**Additional file 12 Fig. S11**. ALKBH5 inhibits tumor growth and metastasis in vivo.

## Data Availability

Supplementary Table 1 and Figs. S1 to S11 are attached.

## Abbreviations

AGO2: Argonaute RISC catalytic component 2
ALKBH5: AlkB homolog 5
Cycloleucine: CL
EIF3a: Eukaryotic translation initiation factor 3 subunit A
EMT: Epithelial-to-mesenchymal transition
FTO: FTO alpha-ketoglutarate dependent dioxygenase
HuR: Human antigen R
LATS2: large tumor suppressor kinase 2
M^6^A: N^6^-methyladenosine
METTL14: Methyltransferase-like 14
METTL3: Methyltransferase-like 3
MiRNAs: MicroRNAs
NSCLC: Non-small cell lung cancer
ShRNA: Short hairpin RNA
SiRNA: Short interfering RNA
TAZ: Transcriptional co-activator with PDZ-binding motif
TEAD: TEA domain family member
UTR: Untranslated region
WTAP: Wilms tumor 1-binding protein
