# Correction: m6A demethylase ALKBH5 inhibits tumor growth and metastasis by reducing YTHDFs-mediated YAP expression and inhibiting miR-107/LATS2–mediated YAP activity in NSCLC

**DOI:** 10.1186/s12943-022-01593-x

**Published:** 2022-06-13

**Authors:** Dan Jin, Jiwei Guo, Yan Wu, Lijuan Yang, Xiaohong Wang, Jing Du, Juanjuan Dai, Weiwei Chen, Kaikai Gong, Shuang Miao, Xuelin Li, Hongliang Sun

**Affiliations:** 1 Clinical Medical Laboratory, Binzhou Medical University Hospital, Binzhou, 256603 People’s Republic of China; 2 Cancer research institute, Binzhou Medical University Hospital, Binzhou, 256603 People’s Republic of China; 3 Department of Thyroid and Breast Surgery, Binzhou Medical University Hospital, Binzhou, 256603 People’s Republic of China; 4 Department of reproductive medicine, Binzhou Medical University Hospital, Binzhou, 256603 People’s Republic of China

**Correction: Mol Cancer 19, 40 (2020)**

**https://doi.org/10.1186/s12943-020-01161-1**

Following publication of the original article [1], the authors identified minor errors in Figs. 1 and 5; specifically:

**Fig. 1:**
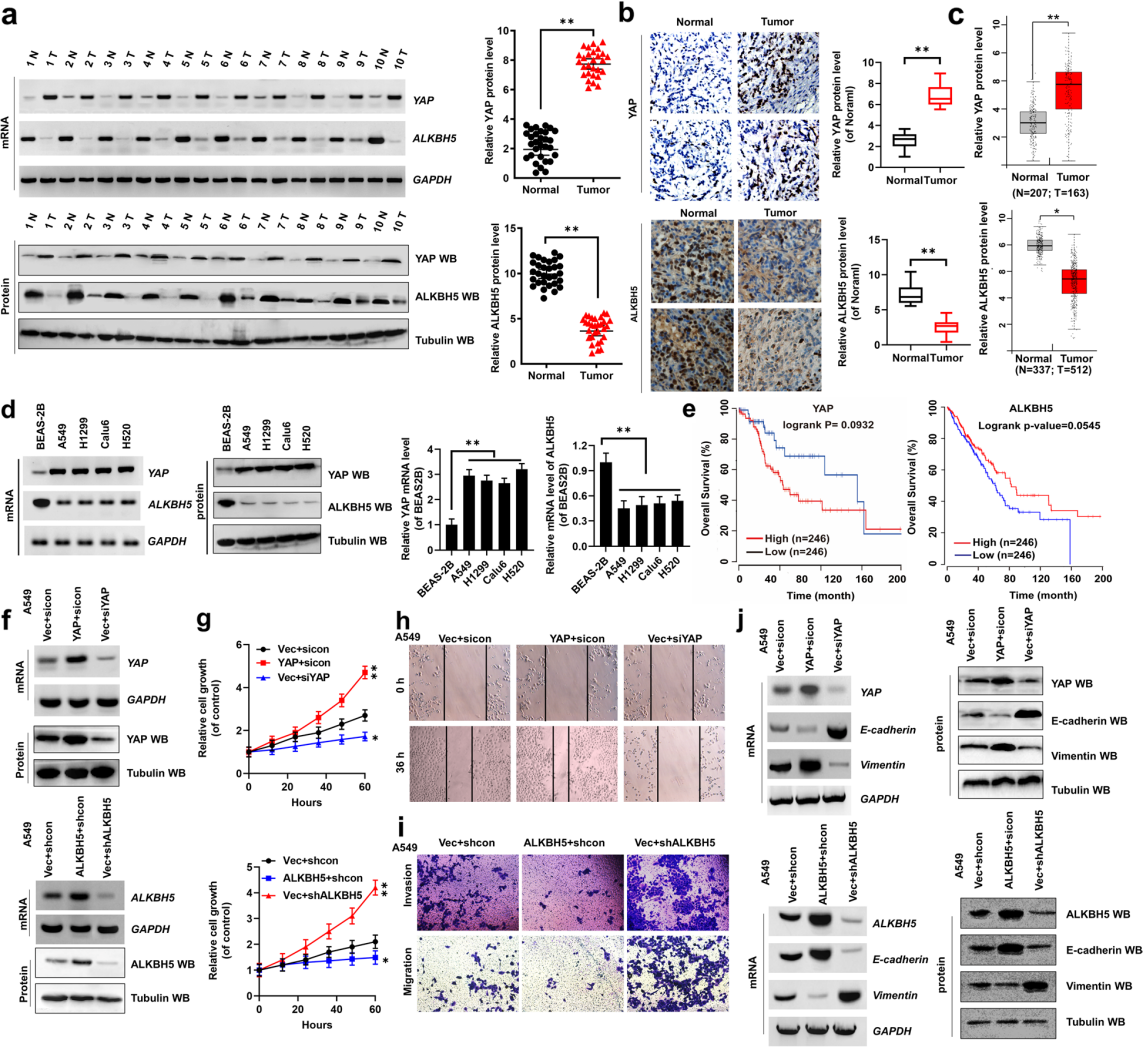
Ectopic expression of YAP and ALKBH5 regulates cell proliferation, invasion, migration, and EMT in NSCLC cells. **a** The mRNA and protein levels of YAP and ALKBH5 were analyzed by RT-PCR and western blot assays in the paired fresh NSCLC tumor cancer tissues (Tumor) and matched adjacent normal tissues (Normal) (left panel, *n* = 10; right panel, *n* = 30). **b** The expressions of YAP and ALKBH5 were analyzed by immunohistochemical (IHC) assay in the human lung cancer tissues and their normal adjacent lung tissues (*n* = 5). **c** The TCGA database indicated that YAP was higher but ALKBH5 was lower in tumor tissues than their normal tissues. **d** The mRNA and protein levels of YAP and ALKBH5 were analyzed by RT-PCR, qPCR and western blot assays in NSCLC cell lines and their control (normal) cell, BEAS-2B. **e** High expression of YAP (*P* = 0.00932) but low expression of ALKBH5 (*P* = 0.00545) were associated with worse prognosis for NSCLC patients. (f-j) A549 cells were transfected with indicated genes of YAP and ALKBH5. **f** The expressions of YAP and ALKBH5 were analyzed by RT-PCR and western blot assays. **g** The cellular growth was analyzed by CCK8 assay. **h** The migration viability was analyzed by scratch assay. (i) The cellular invasion and migration growths were analyzed by transwell assay. **j** The expressions of E-cadherin and Vimentin were analyzed by RT-PCR and western blot assays. Results were presented as mean ± SD of three independent experiments. *P < 0.05 or **P < 0.01 indicates a significant difference between the indicated groups

**Fig. 5:**
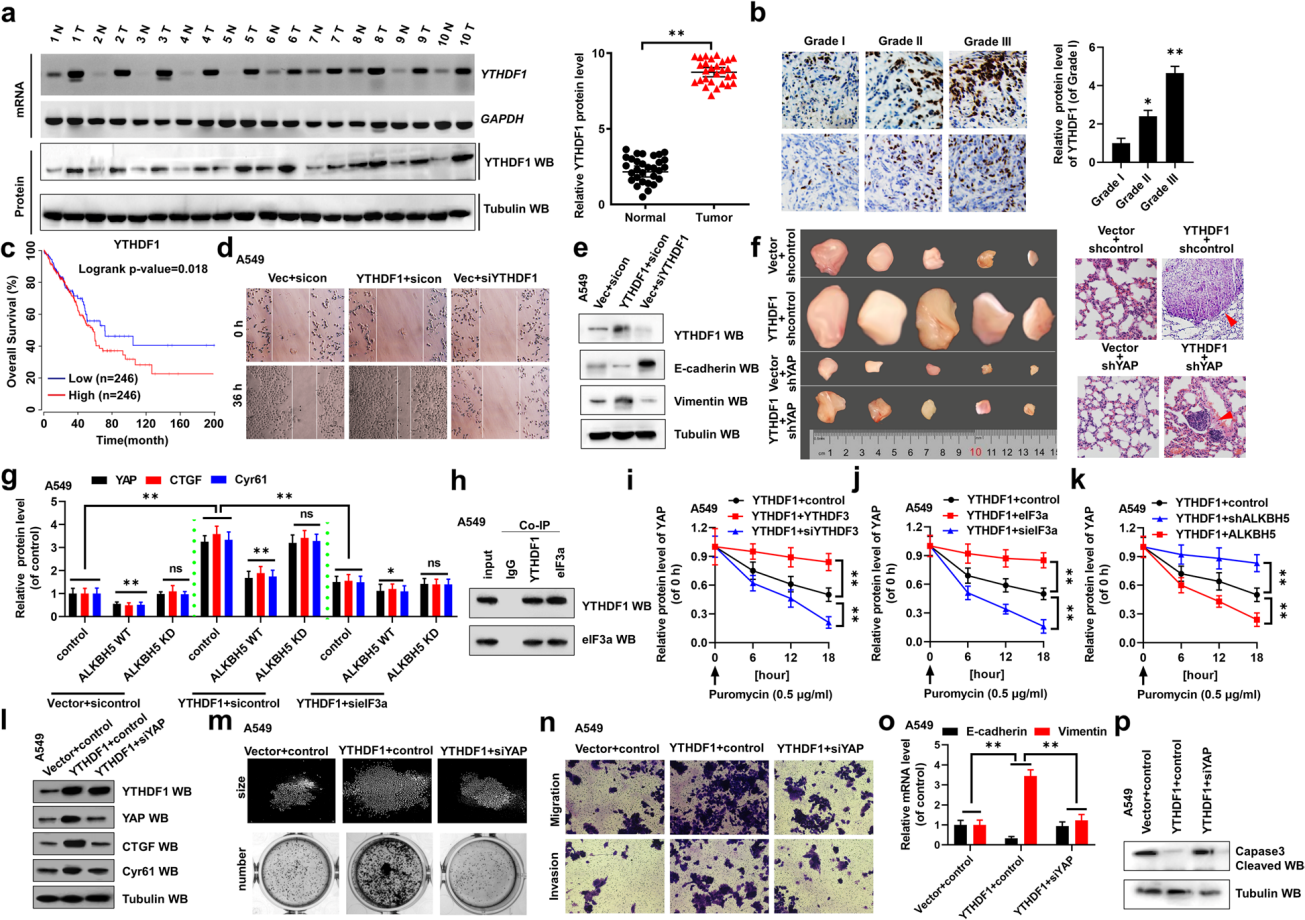
YTHDF1-promoted YAP mRNA translation is regulated by m6A modification and interaction with eIF3a. **a** The mRNA and protein levels of YTHDF1 were analyzed by RT-PCR and western blot assays in paired fresh NSCLC tumor cancer tissues (Tumor) and matched adjacent normal tissues (Normal) (left panel, *n* = 10; right panel, *n* = 30). **b** The expression of YTHDF1 was analyzed by IHC assay in the different grades of lung cancer tissues. **c** High expression of YTHDF1 is associated with worse prognosis for NSCLC patients (*P* = 0.018). (d, e) A549 cells were transfected with indicated genes of YTHDF1. **d** The migration growth was analyzed by scratch assay. **e** The expressions of E-cadherin and Vimentin were analyzed by western blot assay. **f** The xenografted tumors (left panel) and the metastasizing lung tumors (right panel) originated from A549 cells with stable expression of indicated genes constructed by subcutaneous injection. **g** The protein levels of YAP, CTGF and Cyr61 were analyzed in A549 cells determined by WB assay. **h** Co-IPs performed using lysates collected from A549 cells with immunoprecipitation by either YTHDF1 or eIF3a antibodies. (i-k) The protein level of YAP was analyzed by ELISA assay in puromycin treated A549 cells with transfection with indicated genes. (l-p) A549 cell were transfected with indicated genes of YTHDF1 and YAP. **l** The protein levels of YTHDF1, YAP, CTGF and Cyr61 were analyzed by western blot assay. **m** The size and number of colons were analyzed by colony formation assay. **n** The cellular invasion and migration growth were analyzed by transwell assay. **o** The expressions of E-cadherin and Vimentin were analyzed by qPCR assay. **p** The relative of cleaved Caspas-3 was analyzed by western blot assay. Results were presented as mean ± SD of three independent experiments. *P < 0.05 or **P < 0.01 indicates a significant difference between the indicated groups. ns, not significant

Fig. 1b: Incorrect image used to show expression of ALKBH5 Tumor group (bottom right panel); the correct image is now used.

Fig. 5 h: Incorrect image was used for the YTHDF1 band; the correct band is now used.

The corrected figures are given here. The correction does not have any effect on the final conclusions of the paper. The original article has been corrected.
