# Supplementary material for: m6A demethylase ALKBH5 inhibits tumor growth and metastasis by reducing YTHDFs-mediated YAP expression and inhibiting miR-107/LATS2–mediated YAP activity in NSCLC
Source: Mol Cancer. 2020 Feb 27;19:40. doi: 10.1186/s12943-020-01161-1 (PMC7045432; doi:10.1186/s12943-020-01161-1)
Supplement: Supplementary file 1 — Additional file 1 Table S1. Correlation of ALKBH5, YAP, YTHDF1 and YTHDF2 with pathological grades of NSCLC patients. [file 12943_2020_1161_MOESM1_ESM.docx]

**Table S1**. Correlation of individual the protein levels of ALKBH5, YAP, YTHDF1 and YTHDF2 with pathological grades in NSCLC patients determined by IHC assay.

|  | pathological grade | n | mRNA expression | | | p-value |
| --- | --- | --- | --- | --- | --- | --- |
|  |  |  | Low (%) | Median (%) | High (%) |  |
| ALKBH5 | I | 40 | 27 (67.5) | 7 (17.5) | 6 (15.0) | 0.0152 |
|  | II | 43 | 32 (74.4) | 7 (16.3) | 4 (9.3) | 0.0103 |
|  | III | 39 | 36 (92.3) | 2 (5.1) | 1 (2.6) | 0.0045 |
| YAP | I | 46 | 6 (13.1) | 6 (13.1) | 34 (73.8) | 0.0116 |
|  | II | 41 | 3 (7.3) | 4 (9.8) | 34 (82.9) | 0.0104 |
|  | III | 47 | 2 (4.3) | 3 (6.4) | 42 (89.3) | 0.0078 |
| YTHDF1 | I | 45 | 4 (8.9) | 6 (13.3) | 35 (77.8) | 0.0243 |
|  | II | 42 | 3 (7.2) | 4 (9.5) | 35 (83.3) | 0.0106 |
|  | III | 45 | 2 (4.5) | 3 (6.7) | 40 (88.8) | 0.0074 |
| YTHDF2 | I | 44 | 34 (77.3) | 6 (13.6) | 4 (9.1) | 0.0297 |
|  | II | 42 | 35 (85.6) | 3 (7.2) | 3 (7.2) | 0.0109 |
|  | III | 45 | 42 (93.3) | 2 (4.4) | 1 (2.3) | 0.0051 |

Differences between experimental groups were assessed by Student’s t-test or one-way analysis of variance. Data represent mean ± SD. *p<0.05; **p<0.01.
