# Supplementary material for: m6A demethylase ALKBH5 inhibits tumor growth and metastasis by reducing YTHDFs-mediated YAP expression and inhibiting miR-107/LATS2–mediated YAP activity in NSCLC
Source: Mol Cancer. 2020 Feb 27;19:40. doi: 10.1186/s12943-020-01161-1 (PMC7045432; doi:10.1186/s12943-020-01161-1)

**Figure S1.** **Ectopic expression of YAP and ALKBH5 regulates cell proliferation.**

(**a, b**) The mRNA and protein levels of YAP (**a**) and ALKBH5 (**b**) were analyzed by qPCR and western blot assays in the paired NSCLC cancer tissues (T) and their normal adjacent lung tissues (N) (n=10). (**c**) The ALKBH5 protein was analyzed by immunofluorescent staining assay. (**d-m**) A549 and H1299 cells were transfected with indicated genes of YAP and ALKBH5, respectively. (**d-g**) The mRNA levels of YAP (**d, f**) and ALKBH5 (**e, g**) were analyzed by qPCR. (**h, i**) The cellular viability was analyzed by CCK8 assay. (**j, k**) The Ki67 (**j**) and Edu (**k**) positive cells were analyzed by immunofluorescent staining assay. (**l, m**) The protein level of cleaved Caspase 3 was analyzed by western blot assay after transections for 48 h. Results were presented as mean ± SD of three independent experiments. **P* < 0.05 or ***P* < 0.01 indicates a significant difference between the indicated groups. ns, not significant.


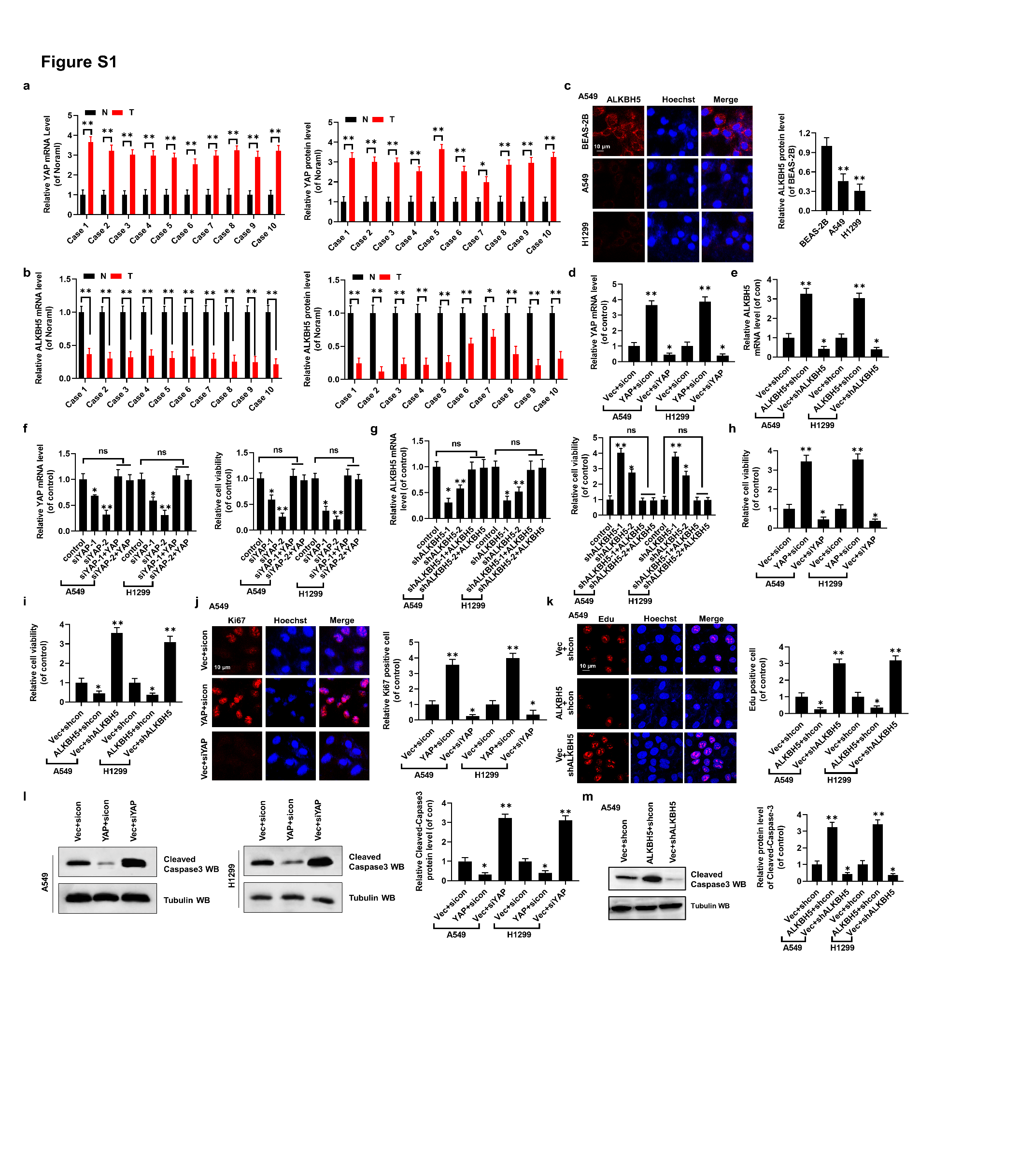

Supplement: Supplementary file 2 — Additional file 2 Fig. S1. Ectopic expression of YAP and ALKBH5 regulates cell proliferation. [file 12943_2020_1161_MOESM2_ESM.docx]
