# Supplementary material for: m6A demethylase ALKBH5 inhibits tumor growth and metastasis by reducing YTHDFs-mediated YAP expression and inhibiting miR-107/LATS2–mediated YAP activity in NSCLC
Source: Mol Cancer. 2020 Feb 27;19:40. doi: 10.1186/s12943-020-01161-1 (PMC7045432; doi:10.1186/s12943-020-01161-1)

**Figure S2.** **Ectopic expression of YAP and ALKBH5 regulates cell migration, invasion and EMT.**

A549 and H1299 cells were transfected with indicated genes of YAP and ALKBH5, respectively. (**a**) The size and number of colons were analyzed by colony formation assay. (**b-e**) The invasion and migration growths were analyzed by scratch (**b, c**) and transwell (**d, e**) assays. (**f, g**) The protein levels of E-cadherin and Vimentin were analyzed by immunofluorescent staining assay. (**h**) The negative correlation between YAP and E-cadherin and positive correlation between YAP and Vimentin analyzed from TCGA database. (**i**) The positive correlation between ALKBH5 and E-cadherin and negative correlation between ALKBH5 and Vimentin analyzed from TCGA database. Results were presented as mean ± SD of three independent experiments. **P* < 0.05 or ***P* < 0.01 indicates a significant difference between the indicated groups.


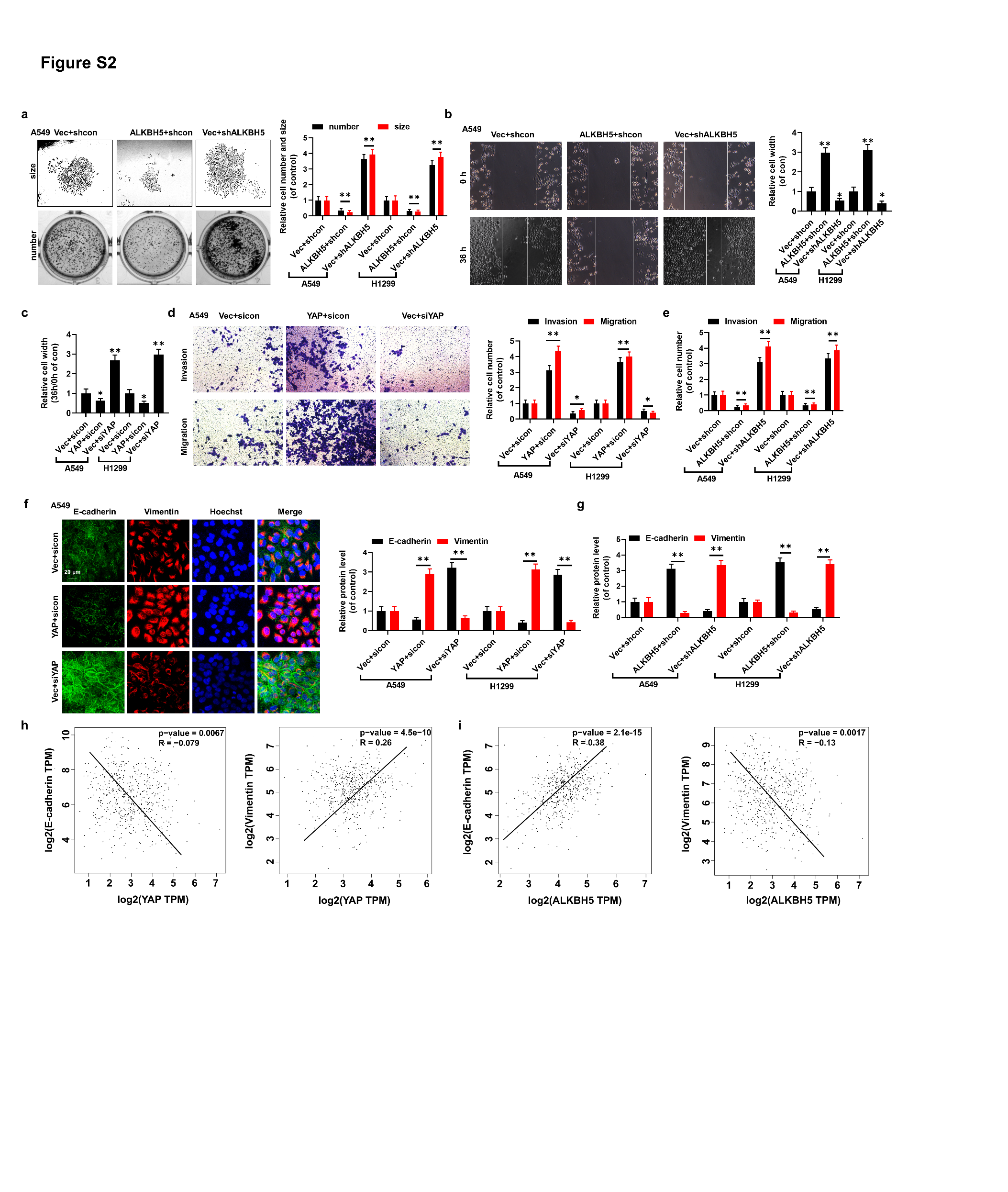

Supplement: Supplementary file 3 — Additional file 3 Fig. S2. Ectopic expression of YAP and ALKBH5 regulates cell migration, invasion and EMT. [file 12943_2020_1161_MOESM3_ESM.docx]
