# Supplementary material for: m6A demethylase ALKBH5 inhibits tumor growth and metastasis by reducing YTHDFs-mediated YAP expression and inhibiting miR-107/LATS2–mediated YAP activity in NSCLC
Source: Mol Cancer. 2020 Feb 27;19:40. doi: 10.1186/s12943-020-01161-1 (PMC7045432; doi:10.1186/s12943-020-01161-1)
Supplement: Supplementary file 4 — Additional file 4 Fig. S3. ALKBH5 controls YAP expression by regulation m6A level in NSCLC. [file 12943_2020_1161_MOESM4_ESM.docx]

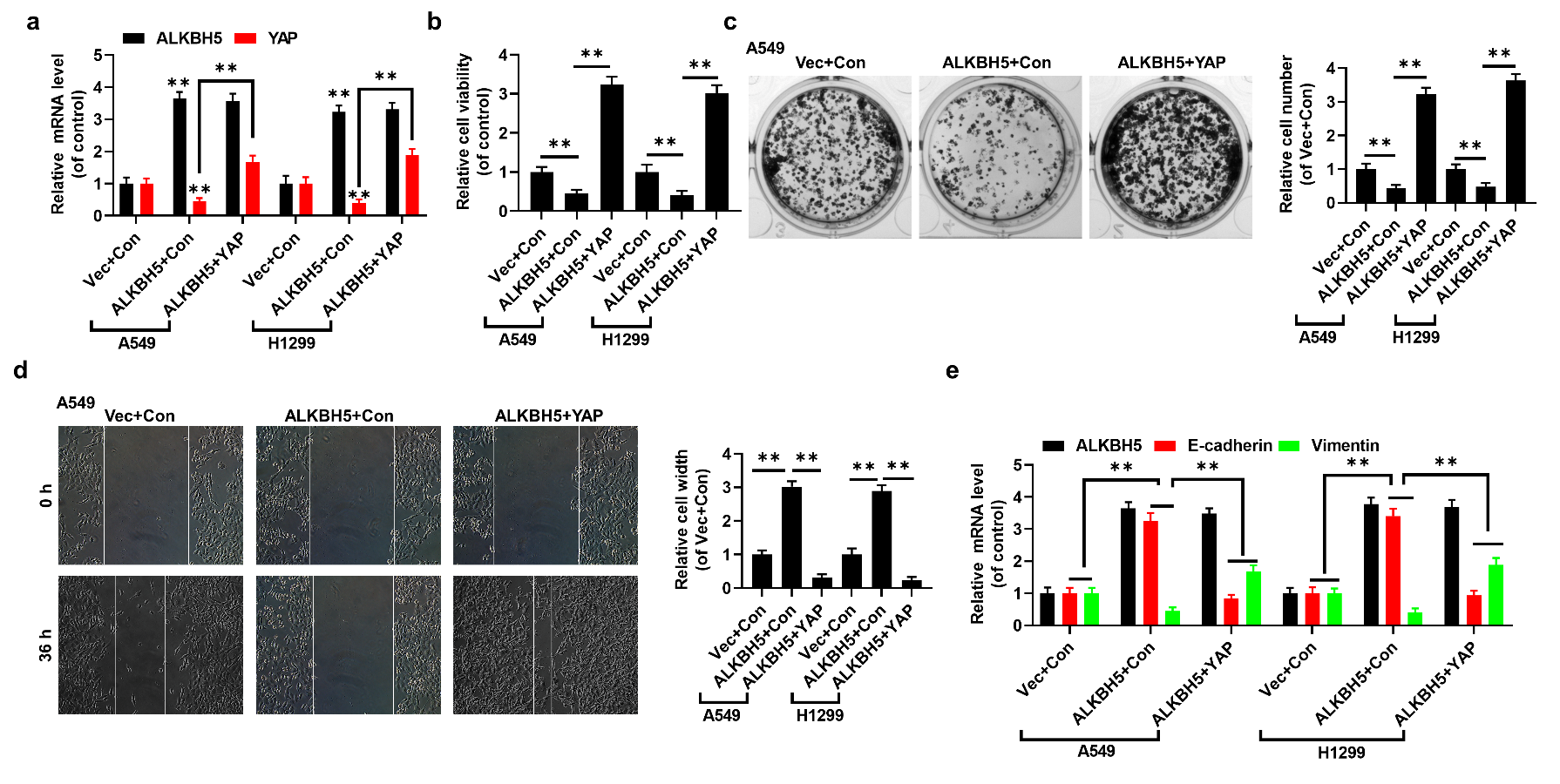
**Figure S3. ALKBH5 inhibits cell growth, migration and EMT by regulation of YAP**

A549 and H1299 cells were transfected with indicated genes of ALKBH5 and YAP, respectively. (**a**) The mRNA levels of ALKBH5 and YAP were analyzed by qPCR assay. (**b**) The cellular viability was analyzed by CCK8 assay. (**c**) Colony formation ability was analyzed by colony formation assay. (**d**) The cellular migration growth was analyzed by scratch assay. (**e**) The mRNA levels of E-cadherin and Vimentin were analyzed by qPCR assay. Results were presented as mean ± SD of three independent experiments. **P* < 0.05 or ***P* < 0.01 indicates a significant difference between the indicated groups.
