# Supplementary material for: m6A demethylase ALKBH5 inhibits tumor growth and metastasis by reducing YTHDFs-mediated YAP expression and inhibiting miR-107/LATS2–mediated YAP activity in NSCLC
Source: Mol Cancer. 2020 Feb 27;19:40. doi: 10.1186/s12943-020-01161-1 (PMC7045432; doi:10.1186/s12943-020-01161-1)

**Figure S4. ALKBH5 controls YAP expression by regulation m^6^A level in NSCLC**

(**a**) The YAP m^6^A levels was detected in human NSCLC patients (n=10) determined by MeRIP-qPCR assay. (**b-e**) The relative m^6^A level of YAP pre-mRNA was detected by MeRIP-qPCR in A549 and H1299 cells with transfection of indicated genes. (**f**) The protein level of YAP was detected in A549 and H1299 cells with transfection of indicated genes. (**g-j**) The mRNA and protein levels of ALKBH5, YAP, CTGF and Cyr61 were detected in A549 and H1299 cells with transfection of ALKBH5 or shALKBH5 by RT-PCR, western blot, qPCR and immunofluorescent staining assays. (**k**) The YAP protein level was analyzed by IHC assay in the different expressions of ALKBH5 from lung cancer tissues. (**l**) The expressions of ALKBH5 and YAP were analyzed by IHC assay in the different grades of NSCLC tumor tissues. (**m**) The mRNA levels of ALKBH5 was detected in H1299 cells with transfection of ALKBH5 WT or KD by qPCR assay. (**n**) The interactions between ALKBH5 WT or KD and YAP pre-mRNA were detected by RIP assay in A549 and H1299 cells. Results were presented as mean ± SD of three independent experiments. **P* < 0.05 or ***P* < 0.01 indicates a significant difference between the indicated groups. ns, not significant.


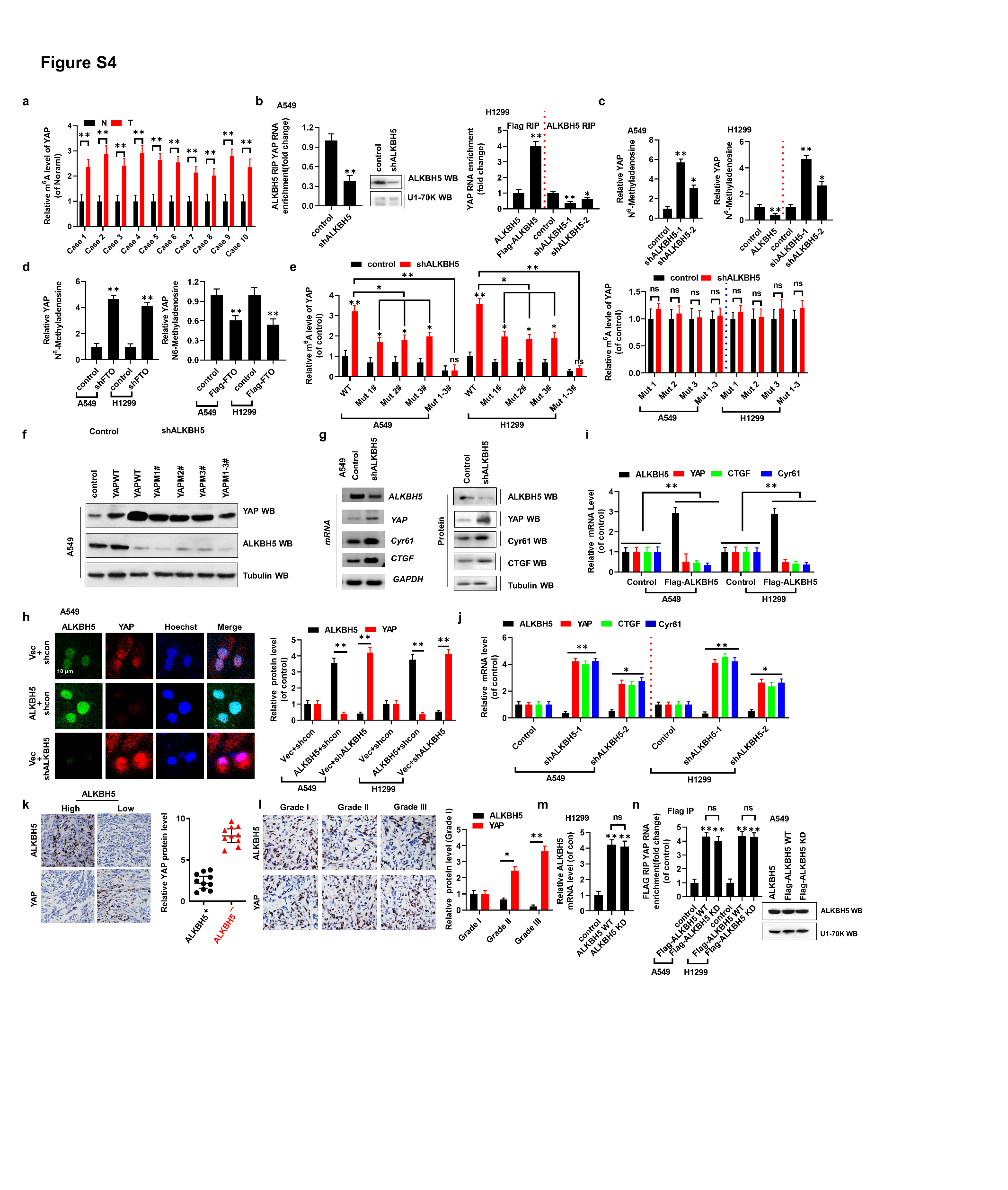

Supplement: Supplementary file 5 — Additional file 5 Fig. S4. ALKBH5 inhibits cell growth, migration and EMT by regulation of YAP. [file 12943_2020_1161_MOESM5_ESM.docx]
