# Supplementary material for: m6A demethylase ALKBH5 inhibits tumor growth and metastasis by reducing YTHDFs-mediated YAP expression and inhibiting miR-107/LATS2–mediated YAP activity in NSCLC
Source: Mol Cancer. 2020 Feb 27;19:40. doi: 10.1186/s12943-020-01161-1 (PMC7045432; doi:10.1186/s12943-020-01161-1)

**Figure S5.** **YTHDF1 and YTHDF2 competitively interacts with YTHDF3**

(**a**) A549 and H1299 cells were transfected with indicated genes then cell lysates were performed RNA pulldown with the baits of YAP mRNA. The relative protein levels of YTHDF1 and YTHDF2 were analyzed by western blot assay. (**b, c**) The nuclear and cytoplasmic distribution of YAP mRNA (**b**) and protein (**c**) were analyzed by qPCR and western blot assays. (**d**) The schematic diagram of MS2 coat protein system to enrich YAP mRNA. (**e**) The interactions between YTHDF1/2/3 and YAP mRNA were detected in H1299 cells with transfection with indicated genes determined by Co-IP assay using the MS2 coat protein system. (**f**) The protein level of YTHDF3 was detected in lysates collected from H1299 cells determined by Co-IP assay with immunoprecipitation with either YTHDF1 or YTHDF2 antibodies. Results were presented as mean ± SD of three independent experiments. **P* < 0.05 or ***P* < 0.01 indicates a significant difference between the indicated groups. ns, not significant.


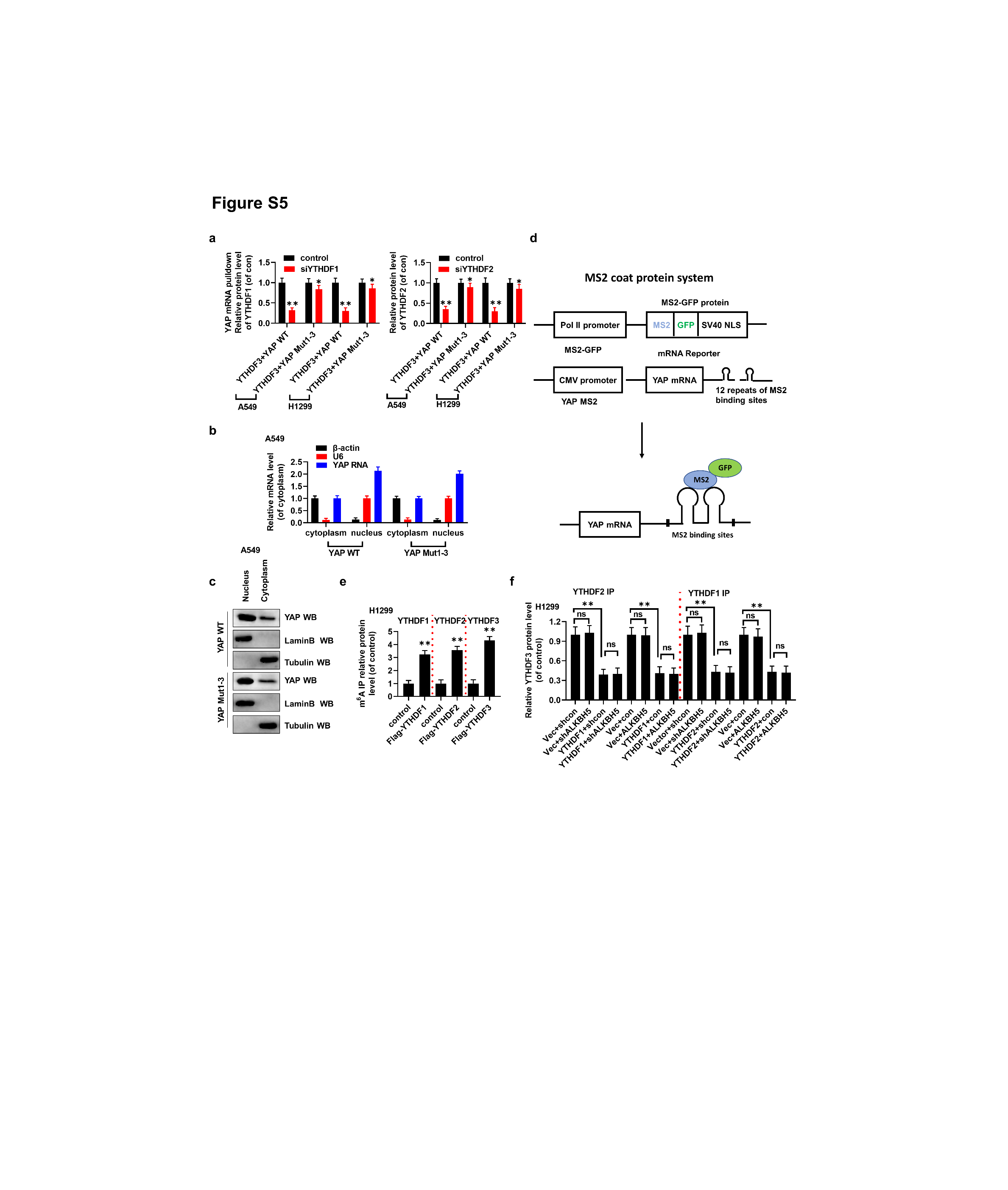

Supplement: Supplementary file 6 — Additional file 6 Fig. S5. YTHDF1 and YTHDF2 competitively interacts with YTHDF3. [file 12943_2020_1161_MOESM6_ESM.docx]
