# Supplementary material for: m6A demethylase ALKBH5 inhibits tumor growth and metastasis by reducing YTHDFs-mediated YAP expression and inhibiting miR-107/LATS2–mediated YAP activity in NSCLC
Source: Mol Cancer. 2020 Feb 27;19:40. doi: 10.1186/s12943-020-01161-1 (PMC7045432; doi:10.1186/s12943-020-01161-1)

**Figure S6. YTHDF2 inhibits tumor growth and metastasis in NSCLC**

(**a**) The mRNA level of YTHDF2 was analyzed in paired tumor tissues (T) and adjacent normal tissues (N) by qPCR (n=10). (**b**) The TCGA database indicated that YTHDF2 expression was lower in tumor tissues than in normal tissues. (**c**) The expressions of YTHDF2 were analyzed in NSCLC cell lines and their control cell, BEAS-2B. (**d, e**) The mRNA level of YTHDF2 was analyzed in A549 and H1299 cells with transfection of indicated genes by qPCR assay. (**f-k**) A549 and H1299 cells were co-transfected with indicated genes of YTHDF2. (**f**) The cellular viability was analyzed by CCK8 assay. (**g**) The protein level of cleaved Caspase 3 was analyzed by western blot assay. (**h**) The Ki67 positive cells were analyzed immunofluorescent staining assay. (**i, j**) The cellular invasion and migration growth were analyzed by scratch (**i**) and transwell (**j**) assays. (**k**) The mRNA levels of E-cadherin and Vimentin were analyzed by qPCR. (**l**) The positive correlation between YTHDF2 and E-cadherin and negative correlation between YTHDF2 and Vimentin analyzed from TCGA database. Results were presented as mean ± SD of three independent experiments. **P* < 0.05 or ***P* < 0.01 indicates a significant difference between the indicated groups.


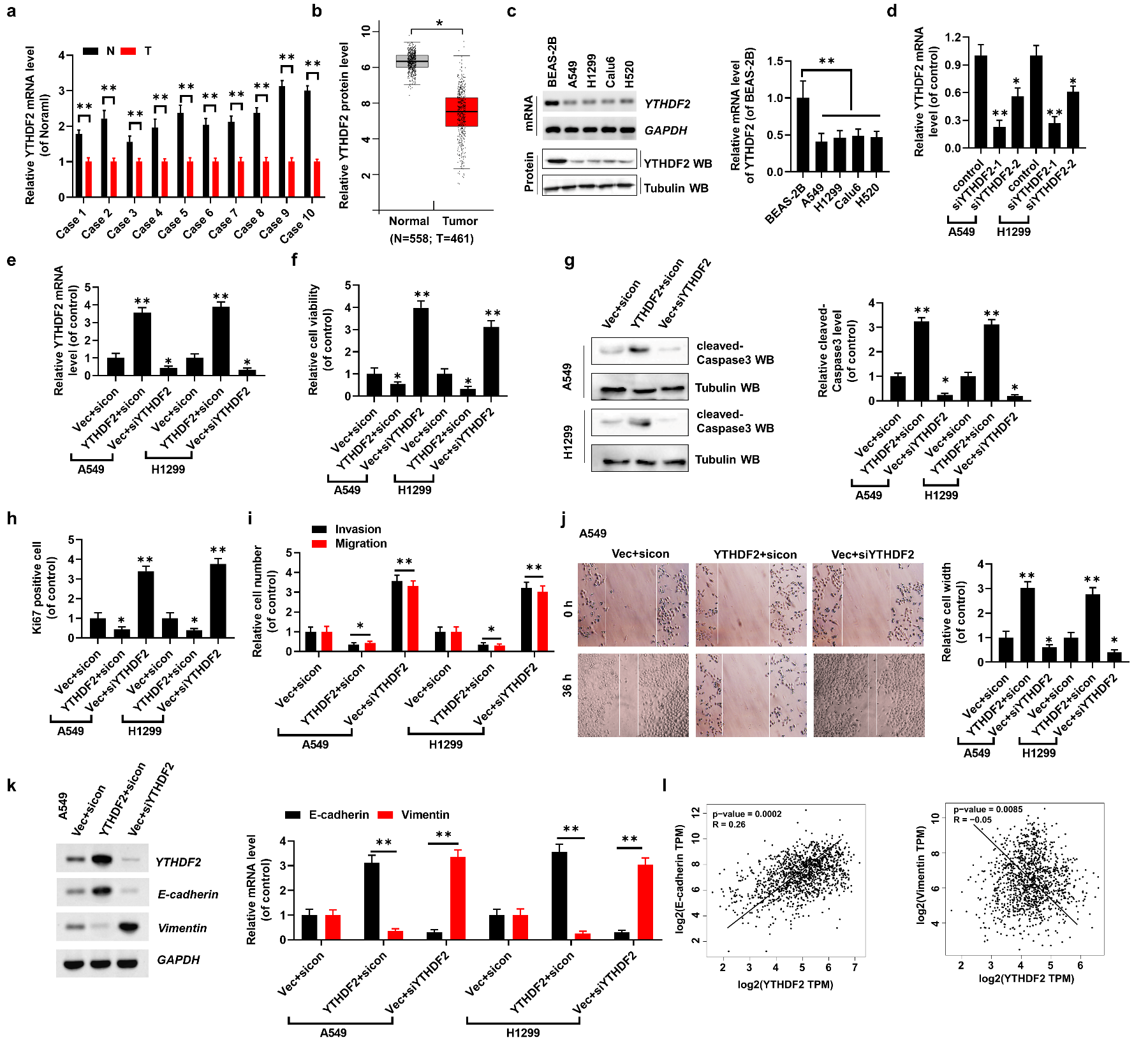

Supplement: Supplementary file 7 — Additional file 7 Fig. S6. YTHDF2 inhibits tumor growth and metastasis in NSCLC. [file 12943_2020_1161_MOESM7_ESM.docx]
