# Supplementary material for: m6A demethylase ALKBH5 inhibits tumor growth and metastasis by reducing YTHDFs-mediated YAP expression and inhibiting miR-107/LATS2–mediated YAP activity in NSCLC
Source: Mol Cancer. 2020 Feb 27;19:40. doi: 10.1186/s12943-020-01161-1 (PMC7045432; doi:10.1186/s12943-020-01161-1)

**Figure S7. YTHDF2-facilitated decay of *YAP* mRNA is mediated by AGO2 system**

(**a**) The mRNA levels of YAP, CTGF and Cyr61 were detected by qPCR in H1299 with transfection into indicated genes. (**b-d**) The Actinomycin (AD)-treated A549 and H1299 cells were transfected into the indicated genes of YTHDF2. The mRNA level of YAP was detected by qPCR (**b, c**) and fluorescence in situ hybridization (FISH) (**d**) assays. (**e-g**) The AD-treated A549 and H1299 cells were respectively transfected the indicated genes. The mRNA levels of YAP, CTGF and Cyr61 were analyzed by qPCR, respectively. (**h-p**) A549 and H1299 cell were co-transfected with indicated genes of YTHDF2 and YAP, respectively. (**h, i**) The mRNA levels of YAP, CTGF and Cyr61 were analyzed by qPCR (**h**) and RT-PCR (**i**) assays. (**j**) The cellular viability was analyzed by CCK8 assay. (**k**) The Ki67 positive cells were analyzed immunofluorescent staining assay. (**l**) The number and size of clones were analyzed by clone formation assay. (**m, n**) The migration and invasion viabilities were analyzed by scratch (**m**) and transwell (**n**) assays. (**o**) The expressions of E-cadherin and Vimentin were analyzed by western blot assay. (**p**) The relative of cleaved Caspas-3 (Caspas-3-Cl) was analyzed by western blot assay. Results were presented as mean ± SD of three independent experiments. **P* < 0.05 or ***P* < 0.01 indicates a significant difference between the indicated groups.


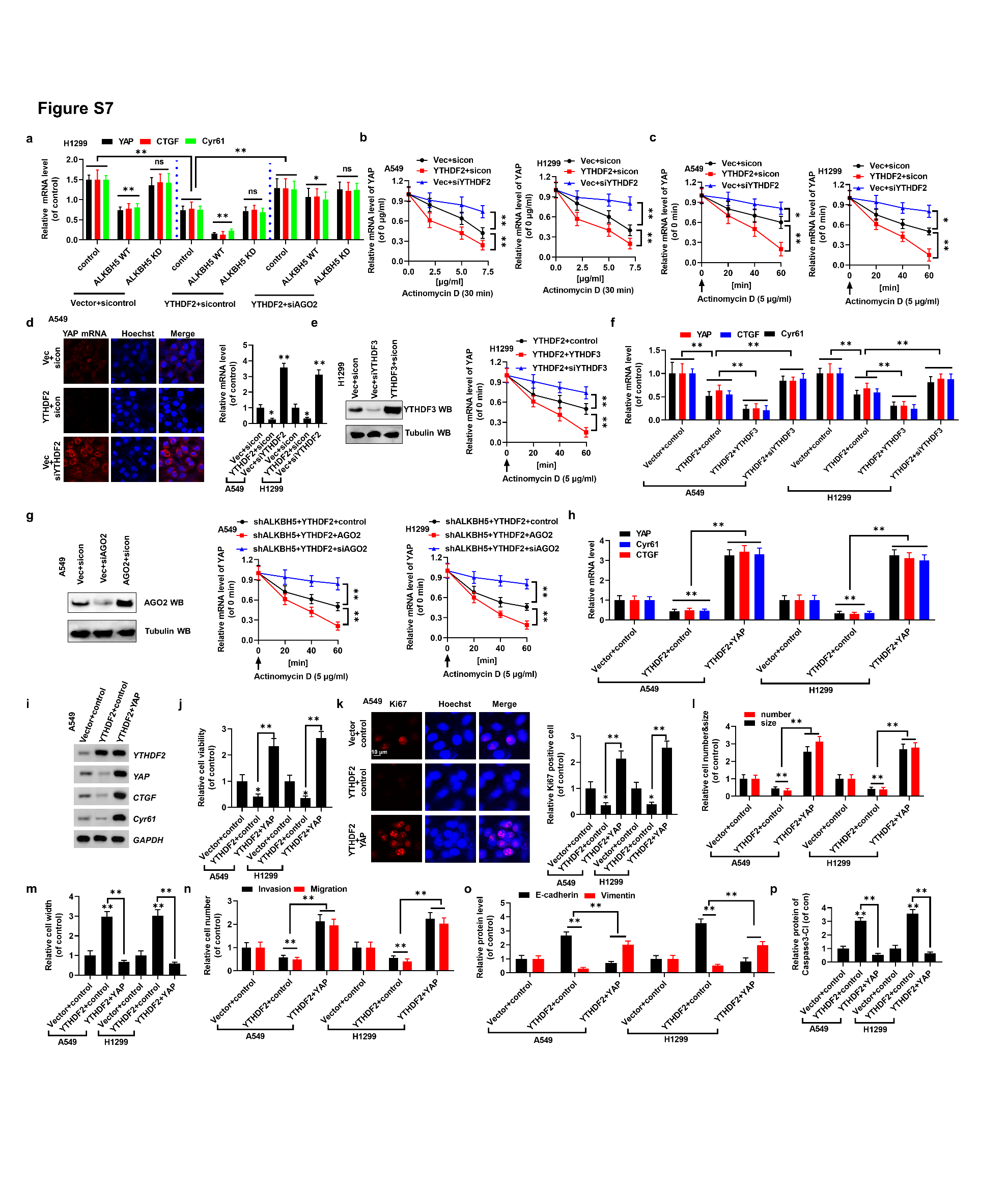

Supplement: Supplementary file 8 — Additional file 8 Fig. S7. YTHDF2-facilitated decay of YAP mRNA is mediated by AGO2 system. [file 12943_2020_1161_MOESM8_ESM.docx]
