# Supplementary material for: m6A demethylase ALKBH5 inhibits tumor growth and metastasis by reducing YTHDFs-mediated YAP expression and inhibiting miR-107/LATS2–mediated YAP activity in NSCLC
Source: Mol Cancer. 2020 Feb 27;19:40. doi: 10.1186/s12943-020-01161-1 (PMC7045432; doi:10.1186/s12943-020-01161-1)

**Figure S8. YTHDF1 promotes tumor growth and metastasis in NSCLC**

(**a**) The mRNA level of YTHDF1 was analyzed by qPCR in paired tumor tissues (T) and adjacent normal tissues (N) by qPCR (n=10). (**b**) The TCGA database indicated that YTHDF1 was higher in tumor tissues than in normal tissues. (**c**) The expressions of YTHDF1 were analyzed in NSCLC cell lines and their control cell, BEAS-2B. (**d, e**) The mRNA level of YTHDF1 was analyzed in A549 and H1299 cells with transfection of the indicated genes determined by qPCR (**d**) and RT-PCR (**e**) assays. (**f-l**) A549 and H1299 cells were transfected with indicated genes of YTHDF1. (**f, g**) The cellular viability (**f**) and growth (**g**) were analyzed by CCK8 assay. (**h**) The Ki67 positive cells were analyzed immunofluorescent staining assay. (**i, j**) The migration and invasion viabilities were analyzed by scratch (**i**) and transwell (**j**) assays. (**k**) The mRNA levels of E-cadherin and Vimentin were analyzed by RT-PCR and qPCR assays. (**l**) The protein level of cleaved Caspase 3 was analyzed by western blot assay. (**m**) The negative correlation between YTHDF1 and E-cadherin and positive correlation between YTHDF1 and Vimentin analyzed from TCGA database. (**n**) The mRNA levels of YTHDF1 and YAP were detected by qPCR in A549 cells with stable expression of indicated genes. (**o, p**) The weight (**o**) and dimension (**p**) of tumors from the xenografted A549 cell tumors with stable expression of indicated genes were measured at regular intervals *in vivo*. (**q**) The overall survival (OS) curves of the mice with the transfected of A549 cells with stable expression of indicated genes. Results were presented as mean ± SD of three independent experiments. **P* < 0.05 or ***P* < 0.01 indicates a significant difference between the indicated groups.


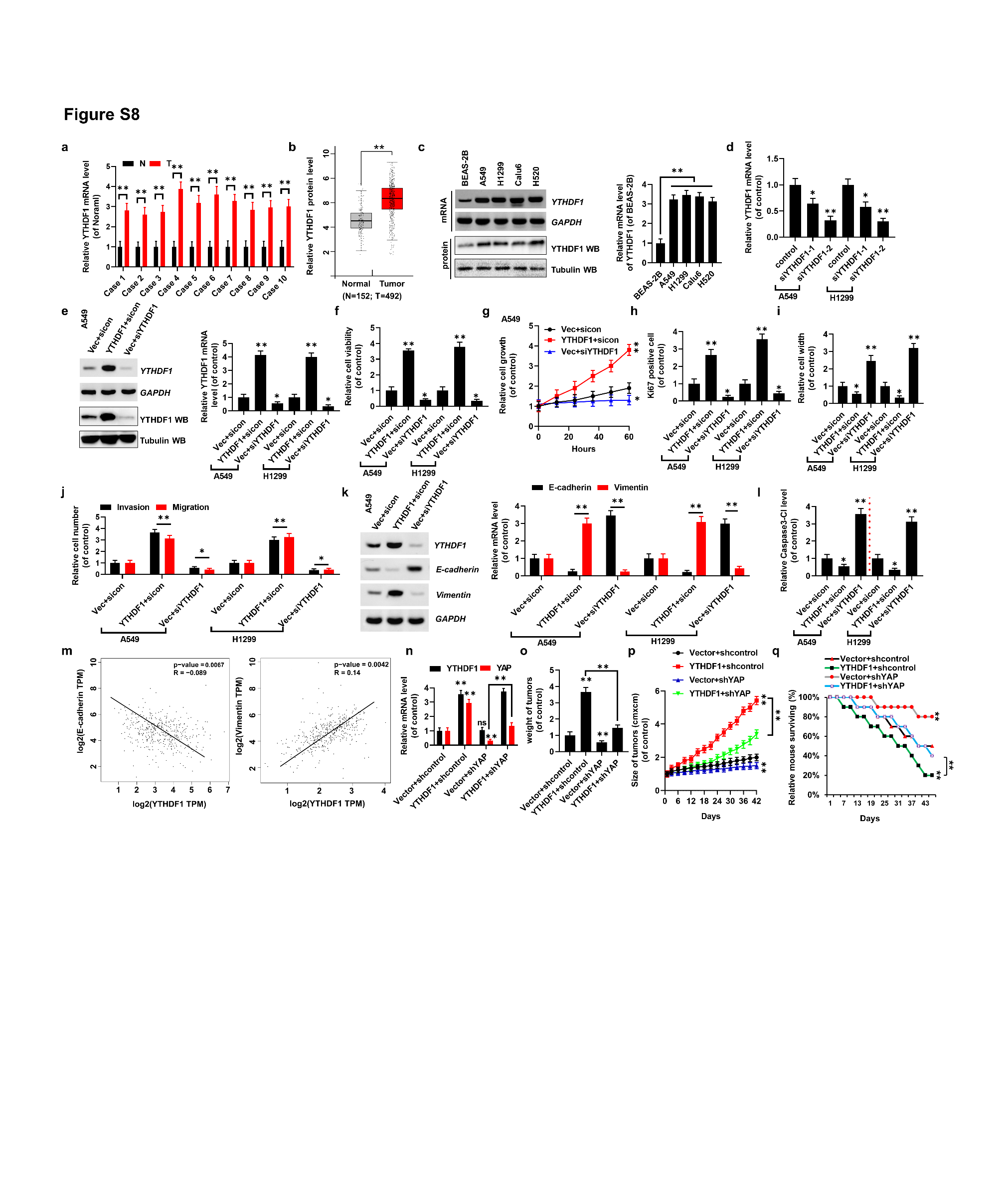

Supplement: Supplementary file 9 — Additional file 9 Fig. S8. YTHDF1 promotes tumor growth and metastasis in NSCLC. [file 12943_2020_1161_MOESM9_ESM.docx]
