# Supplementary material for: m6A demethylase ALKBH5 inhibits tumor growth and metastasis by reducing YTHDFs-mediated YAP expression and inhibiting miR-107/LATS2–mediated YAP activity in NSCLC
Source: Mol Cancer. 2020 Feb 27;19:40. doi: 10.1186/s12943-020-01161-1 (PMC7045432; doi:10.1186/s12943-020-01161-1)

**Figure S9. YTHDF1-promoted *YAP* mRNA translation is regulated by eIF3a**

(**a**) The protein levels of YAP, CTGF and Cyr61 were detected in H1299 with transfection into indicated genes by western blot assay. (**b-h**) The protein level of YAP was detected by ELISA and immunofluorescent staining assays in Puromycin treated A549 and H1299 cells with transfection into indicated genes. (**i-q**) A549 and H1299 cell were transfected with indicated genes YTHDF1 and YAP, respectively. (**i**) The protein levels of YAP, CTGF and Cyr61 were analyzed by western blot assay. (**j, k**) The cellular growth and viability were analyzed by CCK8 assay. (**l**) The Ki67 positive cells were analyzed immunofluorescent staining assay. (**m**) The size and number of clones were analyzed by clone formation assay. (**n, o**) The migration and invasion viabilities were analyzed by scratch (**n**) and transwell (**o**) assays. (**p**) The expressions of E-cadherin and Vimentin were analyzed by qPCR assay. (**q**) The relative of cleaved Caspas-3 (Caspas-3-Cl) was analyzed by western blot assay. Results were presented as mean ± SD of three independent experiments. **P* < 0.05 or ***P* < 0.01 indicates a significant difference between the indicated groups.


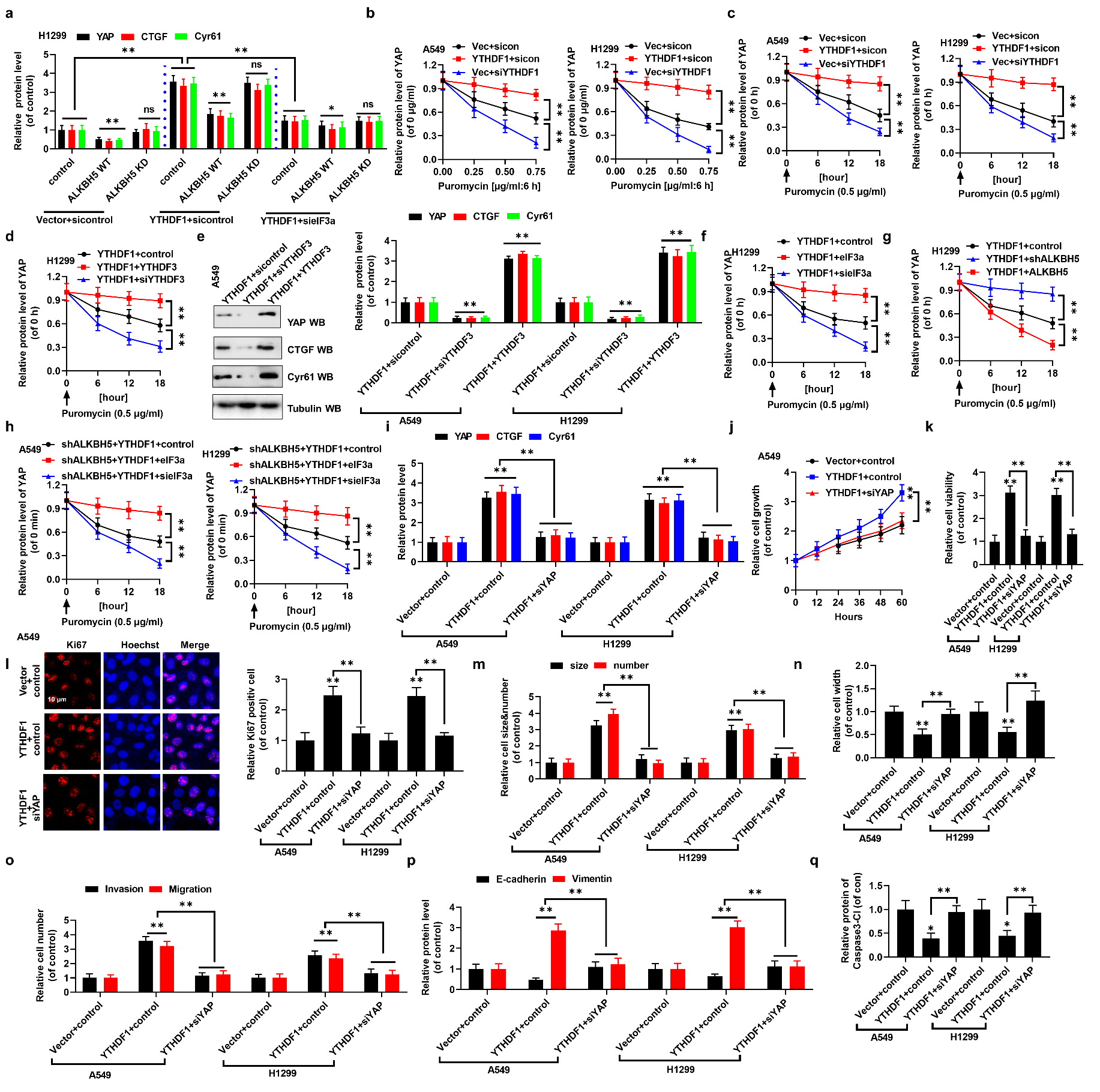

Supplement: Supplementary file 10 — Additional file 10 Fig. S9. YTHDF1-promoted YAP mRNA translation is regulated by eIF3a. [file 12943_2020_1161_MOESM10_ESM.docx]
