# Supplementary material for: m6A demethylase ALKBH5 inhibits tumor growth and metastasis by reducing YTHDFs-mediated YAP expression and inhibiting miR-107/LATS2–mediated YAP activity in NSCLC
Source: Mol Cancer. 2020 Feb 27;19:40. doi: 10.1186/s12943-020-01161-1 (PMC7045432; doi:10.1186/s12943-020-01161-1)

**Figure S10. ALKBH5 decreases YAP activity**

(**a-c**) The mRNA and protein levels of ALKBH5, HuR and miR-107 were detected in A549 and H1299 cells with transfection into indicated genes by RT-PCR, western blot and qPCR assays. (**d**) The luciferase reporter activity of the wild-type and mutated LATS2 was detected in H1299 cells. (**e**) qPCR analyzed the RNA levels of miR-107 in the products of A549 and H1299 cells determined by pulldown with biotin. (**f, g**) The RNA level of miR-107 and LAST2 were analyzed by RT-PCR, western blot and qPCR. (**h**) The positive correlations between ALKBH5/HuR and LATS2 analyzed from TCGA database. (**i**) The protein levels of LATS2, YAP and p-YAP were analyzed by western blot assay. (**j**) A549 cells were transfected miR-107 mimics (miR-107-m) and miR-107 inhibitors (miR-107-i), respectively. The protein level and distribution of YAP were analyzed by immunofluorescent staining assay. (**k-n**) A549 and H1299 cells were co-transfected with indicated genes of *ALKBH5* and *miR-107* mimics, respectively. (**k**) The protein levels of CTGF and Cyr 61 were analyzed by western blot assay. (**l**) The cellular viability was analyzed by CCK8 assay. (**m**) The cellular invasion and migration growths were analyzed by transwell assay. (**n**) The expressions of E-cadherin and Vimentin were analyzed by qPCR assays. (**o-r**) A549 and H1299 cells were co-transfected with indicated genes, *ALKBH5* and *LATS2*, respectively. (**o)** The protein levels of CTGF and Cyr 61 were analyzed by western blot assay. (**p**) The cellular viability was analyzed by CCK8 assay. (**q**) The cellular invasion and migration growths were analyzed by transwell assay. (**r**) The expressions of E-cadherin and Vimentin were analyzed by qPCR assay. Results were presented as mean ± SD of three independent experiments. **P* < 0.05 or ***P* < 0.01 indicates a significant difference between the indicated groups. ns, not significant.


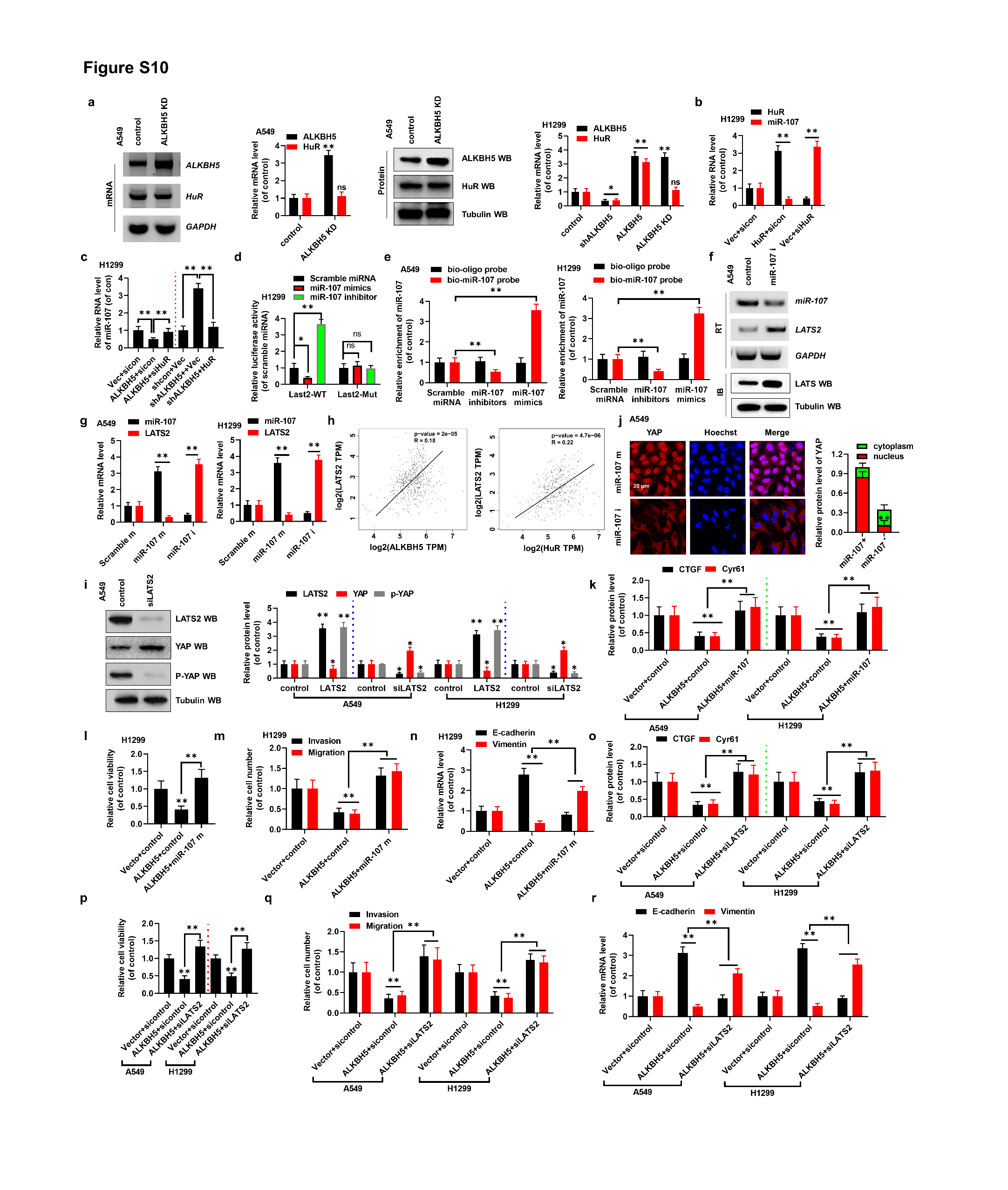

Supplement: Supplementary file 11 — Additional file 11 Fig. S10. ALKBH5 decreases YAP activity. [file 12943_2020_1161_MOESM11_ESM.docx]
