# Supplementary material for: m6A demethylase ALKBH5 inhibits tumor growth and metastasis by reducing YTHDFs-mediated YAP expression and inhibiting miR-107/LATS2–mediated YAP activity in NSCLC
Source: Mol Cancer. 2020 Feb 27;19:40. doi: 10.1186/s12943-020-01161-1 (PMC7045432; doi:10.1186/s12943-020-01161-1)

**Figure S11 ALKBH5 inhibits tumor growth and metastasis *in vivo***

(**a, b**) The protein (**e**) and mRNA (**b**) levels of ALKBH5, YTHDF1, Cyr61 and cleaved Caspase-3 were detected in xenografted A549 cell tumors with stable expression of indicated genes determined by immunohistochemical staining (n=5) and qPCR assays. (**c**) The protein levels of CTGF, Cyr61 and cleaved Caspase-3 were detected in xenografted A549 cell tumors with stable expression of indicated genes determined by western blot assay. (**d-i**) The cycloleucine (CL) and control vehicle-treated A549 and H1299 cells were transfected with indicated genes, respectively. (**d, e**) The mRNA level of ALKBH5 was analyzed by RT-PCR and qPCR assays. (**f**) The m^6^A level of YAP was analyzed by MeRIP-qPCR assay. (**g**) The cellular growth was analyzed by CCK8 assay. (**h**) The cell migration growth was analyzed by scratch assay. (**i**) The expression of E-cadherin and Vimentin were analyzed by qPCR assay. (**j, k**) The protein (**j**) and mRNA (**k**) levels of ALKBH5, YAP, CTGF, Cyr61, Vimentin and E-cadherin were detected in relevant treated tumors with stable expression of indicated genes determined by immunohistochemical staining (n=5) and qPCR assays. Results were presented as mean ± SD of three independent experiments. ***P* < 0.01 indicates a significant difference between the indicated groups. ns, not significant.


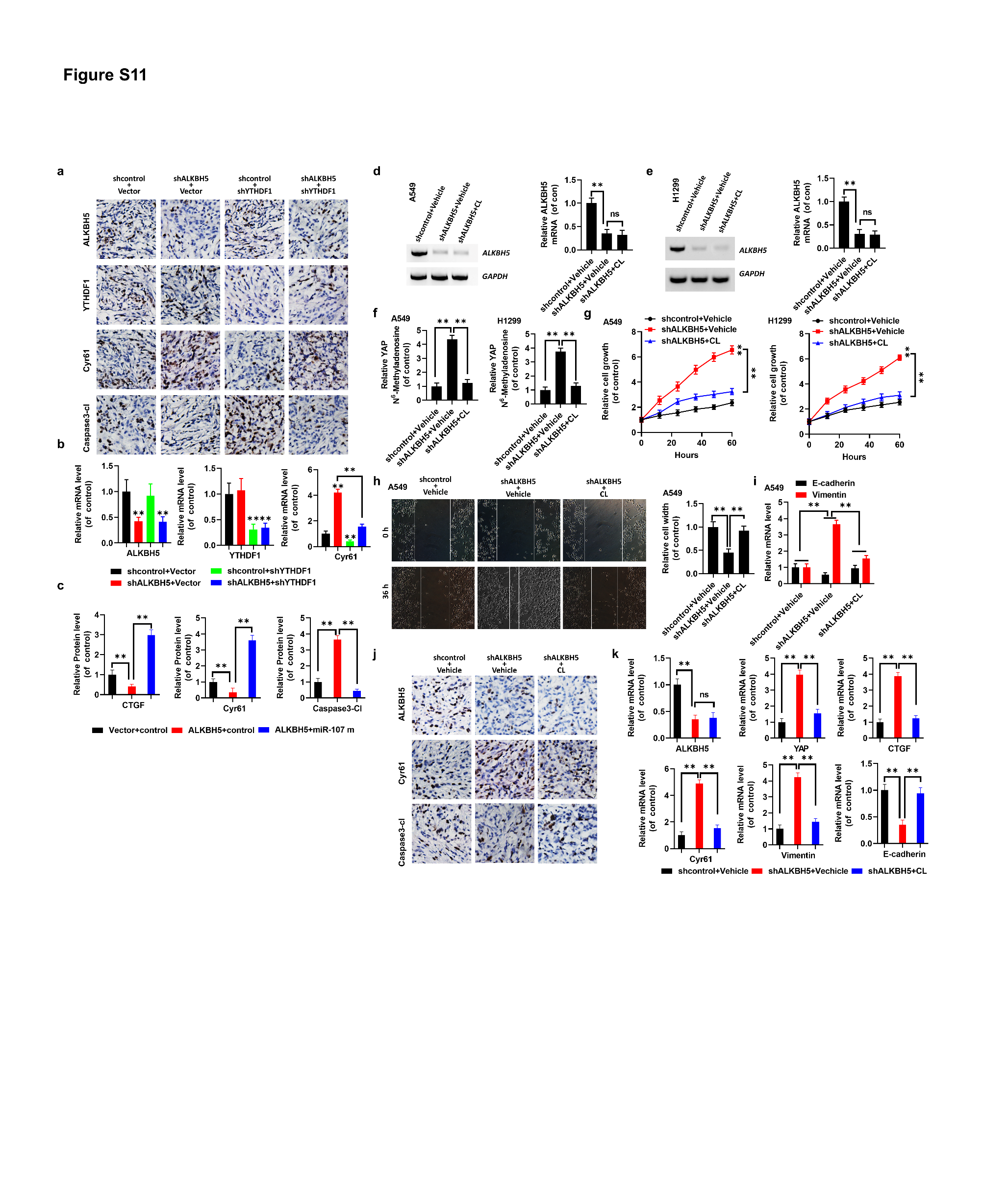

Supplement: Supplementary file 12 — Additional file 12 Fig. S11. ALKBH5 inhibits tumor growth and metastasis in vivo. [file 12943_2020_1161_MOESM12_ESM.docx]
